# Copy number variation of E3 ubiquitin ligase genes in peripheral blood leukocyte and colorectal cancer

**DOI:** 10.1038/srep29869

**Published:** 2016-07-15

**Authors:** Haoran Bi, Tian Tian, Lin Zhu, Haibo Zhou, Hanqing Hu, Yanhong Liu, Xia Li, Fulan Hu, Yashuang Zhao, Guiyu Wang

**Affiliations:** 1 Department of Epidemiology, Public Health College, Harbin Medical University, 157 Baojian Street, Harbin, Heilongjiang, People’s Republic of China; 2 Department of Colorectal Cancer Surgery, The Second Affiliated Hospital of Harbin Medical University, 246 Xuefu Street, Harbin, Heilongjiang, People’s Republic of China; 3 Department of Clinical Laboratory, The Second Affiliated Hospital of Harbin Medical University, 246 Xuefu Street, Harbin, Heilongjiang, People’s Republic of China; 4 College of Bioinformatics Science and Technology, Harbin Medical University, 157 Baojian Street, Harbin, Heilongjiang, People’s Republic of China

## Abstract

Given that E3 ubiquitin ligases (E3) regulate specific protein degradation in many cancer-related biological processes. E3 copy number variation (CNV) may affect the development and prognosis of colorectal cancer (CRC). Therefore, we detected CNVs of five E3 genes in 518 CRC patients and 518 age, gender and residence matched controls in China, and estimated the association between E3 gene CNVs and CRC risk and prognosis. We also estimated their interactions with environmental factors and CRC risk. We find a significant association between the CNVs of *MDM2* and CRC risk (amp *v.s.* wt: odds ratio = 14.37, 95% confidence interval: 1.27, 163.74, *P* = 0.032), while *SKP2* CNVs may significantly decrease CRC risk (del *v.s.* wt: odds ratio = 0.32, 95% confidence interval: 0.10, 1.00, *P* = 0.050). However, we find no significant association between the CNVs of other genes and CRC risk. The only significant gene-environment interaction effects are between *SKP2* CNVs and consumption of fish and/or fruit (*P* = 0.014 and *P* = 0.035) and between *FBXW7* CNVs and pork intake (*P* = 0.040). Finally, we find marginally significant association between *β-TRCP* CNVs and CRC prognosis (amp *v.s.* wt, hazard ratio = 0.42, 95% confidence interval: 0.19, 0.97, *P* = 0.050).

Colorectal cancer (CRC) is the second most common cancer in women and the third most common in men worldwide^1^. In 2012, the World Health Organization estimated that about 1,360,000 new CRC cases occurred worldwide. In addition, 694,000 deaths from CRC were estimated worldwide, accounting for 8.5% of all cancer deaths, and making CRC the fourth most common cause of death from cancer^2^. Although the relative 5-year survival rate of European CRC patients increased between 1930 and 2010^3^, that 5-year survival rate was only 30–65% worldwide^4^.

Genetic susceptibility has a well-established role in the etiology of CRC^5^,^6^. Accumulating evidence supports the hypothesis that copy number variation (CNV) is a molecular biomarker for CRC risk and prognosis^7^. DNA CNVs, as structural variants, can be small: microscopic or submicroscopic; or they can be large: deletions, duplications or insertions, often larger than 1 kb^8^,^9^.

CNVs in the E3 ubiquitin ligases (E3) of the ubiquitin-proteasome system (UPS) have been associated with CRC risk and prognosis^10^,^11^,^12^. E3 plays a critical role in the specific protein degradation of UPS, which has an essential regulatory role in cell cycle progression, cell proliferation, differentiation, apoptosis, angiogenesis and cell signaling pathways^13^,^14^. The two main subfamilies of E3s are RING and HECT domain containing E3s^15^. As members of RING E3s, *FBXW7, MDM2, SKP2* and *β-TRCP* have been associated with abnormal expression in some malignancies including blood, breast, colon and prostate^16^,^17^,^18^,^19^. As a HECT E3 ligase, *NEDD4-1* was also proposed to play a vital role in a number of human cancers, including CRC^20^,^21^.

*FBXW7* serves as a tumor suppressor gene (*p53*-dependent)^22^, and loss of *FBXW7* has been associated with CRC risk and poor prognosis^23^. *MDM2*, functioning as an oncogene, is amplified in approximately one-third of all human carcinomas including CRC^24^. Increased expression of *SKP2* has been significantly associated with poor tumor differentiation and poor prognosis in CRC^18^. Overexpression of *β-TRCP* has also been observed in many tumors, such as CRC^25^, pancreatic cancer^26^, and breast cancer^27^. *NEDD4-1*, as a HECT E3 ligase, is highly expressed in both colorectal and gastric tumor tissues^20^.

Studies of the CNVs of *FBXW7, MDM2, SKP2, β-TRCP* and *NEDD4-1* genes are mainly limited to intestinal cancer cell lines and clinical pathological tissues^10^,^11^,^12^,^19^,^23^,^25^,^28^,^29^,^30^,^31^,^32^. In addition, most studies focus on gene expression; the impact of germline CNVs of these five genes on CRC risk and prognosis are not fully understood. Therefore, we conducted a case-control study to explore associations between the CNVs of *FBXW7, MDM2, SKP2, β-TRCP* and *NEDD4-1* genes and CRC risk. We also followed up with cases to study the association between the CNVs of these five genes and CRC prognosis in China.

## Results

### Characteristics of study subjects

The basic characteristics of the 518 CRC patients and the 518 gender, age, and residence matched controls are summarized in Table 1. However, 32 pairs of our samples were unable to be genotyped in one of the five genes, so gender was not equally distributed in cases and controls (*P* = 0.002). Education (*P* < 0.001), occupation (*P* < 0.001) and family history of other cancers (*P* < 0.001) were also differently distributed in cases and controls. Of the 518 CRC cases, 262 (57.8%) were colon cancer, 191 (42.2%) were rectal cancer. Gender, occupation, education and family history of cancer were adjusted in the following analysis.

### Copy number variation and CRC risk

The *FBXW7, MDM2, SKP2, β-TRCP,* and *NEDD4-1* CNVs were in Hardy-Weinberg equilibrium in all controls. Table 2 shows the CNV frequencies of the five genes and the relationship between the CNVs of the five genes and CRC risk.

We observed significant associations between *MDM2* amplification and increased CRC risk (amp *v.s.* wt: OR_adjusted_ = 14.37, 95% CI: 1.27, 163.74, *P* = 0.032; amp *v.s.* del + wt: OR_adjusted_ = 14.40, 95% CI: 1.26, 164.81, *P* = 0.032). We observed marginally significant association between *SKP2* deletions and CRC risk (del *v.s.* wt: OR_adjusted_ = 0.32, 95% CI: 0.10, 1.00, *P* = 0.050). While there was no significant association between *SKP2* amplification and CRC risk in the amp *v.s.* del + wt model (amp *v.s.* del + wt model: OR = 0.33, 95% CI: 0.11, 1.02, *P* = 0.055). However, we observed no significant associations between *FBXW7, β-TRCP* or *NEDD4-1* CNVs and CRC risk.

### Abnormal copy number additive model and CRC risk

In the abnormal copy number additive model, *MDM2* CNVs are significantly associated with increased CRC risk (del + amp *v.s.* wt: OR_adjusted_ = 6.35, 95% CI: 1.67, 24.19, *P* = 0.007). In the additive models, *SKP2* CNVs also significantly decrease CRC risk (del + amp *v.s.* wt: OR_adjusted_ = 0.32, 95% CI: 0.14, 0.72, *P* = 0.006).

### Gene-environment interactions on CRC risk

We find a significant synergistic interaction effect between *SKP2* CNVs and fruit consumption (amp *v.s.* del + wt: OR_i_ = 13.89, 95% CI: 1.20, 160.57, *P* = 0.035) (Table 3). In addition, there is a significant interaction effect between the amplification of *SKP2* and roughage consumption (≥50 g/week) (amp *v.s.* del + wt: OR_eg_ = 0.18, 95% CI: 0.03, 0.99). We also find significant interaction effects between the amplification of *FBXW7* and consumption of roughage (≥50 g/week) or fish (>once/week) (OR_eg_ = 0.37, 95% CI: 0.15, 0.91 and OR_eg_ = 0.25, 95% CI: 0.07, 0.94, respectively). There were also significant interaction effects between the amplification of *NEDD4-1* and consumption of refined grains (>250 g/week) (OR_eg_ = 2.83, 95% CI: 1.02, 7.88), Chinese pickled sour cabbage (>twice/month) (OR_eg_ = 3.59, 95% CI: 1.23, 10.48), and fatty meats (OR_eg_ = 3.60, 95% CI: 1.27, 10.19).

### Gene-environment interactions in abnormal copy number additive model

We observed significant synergistic interactions between *SKP2* del + amp genotype and fish intake on CRC risk (del + amp *v.s.* wt: OR_i_ = 13.62, 95% CI: 1.70, 109.36, *P* = 0.014) (Table 3). In addition, We also observed significant interaction effects between the del + amp genotype of *SKP2* and roughage consumption (≥50 g/week), or fruit (≥twice/week) consumption (del + amp *v.s.* wt: OR_eg_ equal to 0.13 (95% CI: 0.04, 0.44) and 0.33 (95% CI: 0.12, 0.96), respectively). We also find significant interaction effects between the del + amp genotype of *MDM2* and consumption of refined grains (>250 g/week) (OR_eg_ = 5.44, 95% CI: 1.03, 28.86), fatty meats (OR_eg_ = 8.55, 95% CI: 1.22, 59.75), eggs (>3/week) (OR_eg_ = 7.33, 95% CI: 1.57, 34.30), Chinese pickled sour cabbage (>twice/month) (OR_eg_ = 27.61, 95% CI: 2.12, 259.81) and leftovers (>3 times/week) (OR_eg_ = 26.67, 95% CI: 2.62, 271.60). Moreover, we observed a significant interaction between *FBXW7* CNVs and pork consumption (>250 g/week) (del + amp *v.s.* wt: OR_i_ = 3.13, 95% CI: 1.06, 9.41, *P* = 0.040). We find significant interaction effects between the del + amp genotype of *FBXW7* and consumption of refined grains (>250 g/day), fatty meats and physical exercise (OR_eg_ = 2.81 (95% CI: 1.52, 6.86), OR_eg_ = 2.30 (95% CI: 1.03, 5.11) and OR_eg_ = 0.06 (95% CI: 0.01, 0.31), respectively). Finally, we also find significant interaction effects between the del + amp genotype of *NEDD4-1* and consumption of refined grains (>250 g/day), fatty meats and physical exercise (OR_eg_ = 2.75 (95% CI: 1.00, 7.55) OR_eg_ = 3.30 (95% CI: 1.21, 9.14) and OR_eg_ = 0.06 (95% CI: 0.01, 0.31), respectively).

### Copy number variations and CRC prognosis

323 patients completed the follow-up (Table 4). Of the 323 patients, 186 (57.9%) patients didn’t receive chemotherapy, 45 (14.0%) patients received FOXFOX4-based chemotherapy, 22 (6.9%) received XELOX-based chemotherapy, 44 (13.7%) received LCF-based chemotherapy, 6 (1.9%) received 5-Fu-based chemotherapy and 18 (5.6%) received other chemotherapy treatments after surgery. The mean overall survival (OS) of CRC patients was 75.35 ± 2.26 months. The CEA and CA19-9 level before surgery, Dukes stage, pathological type and metastasis were adjusted for in the analysis of *FBXW7, MDM2, SKP2, β-TRCP* and *NEDD4-1* CNVs and CRC prognosis, due to their significant association with CRC prognosis in the univariate Cox proportional hazards regression.

**Figure 1:**
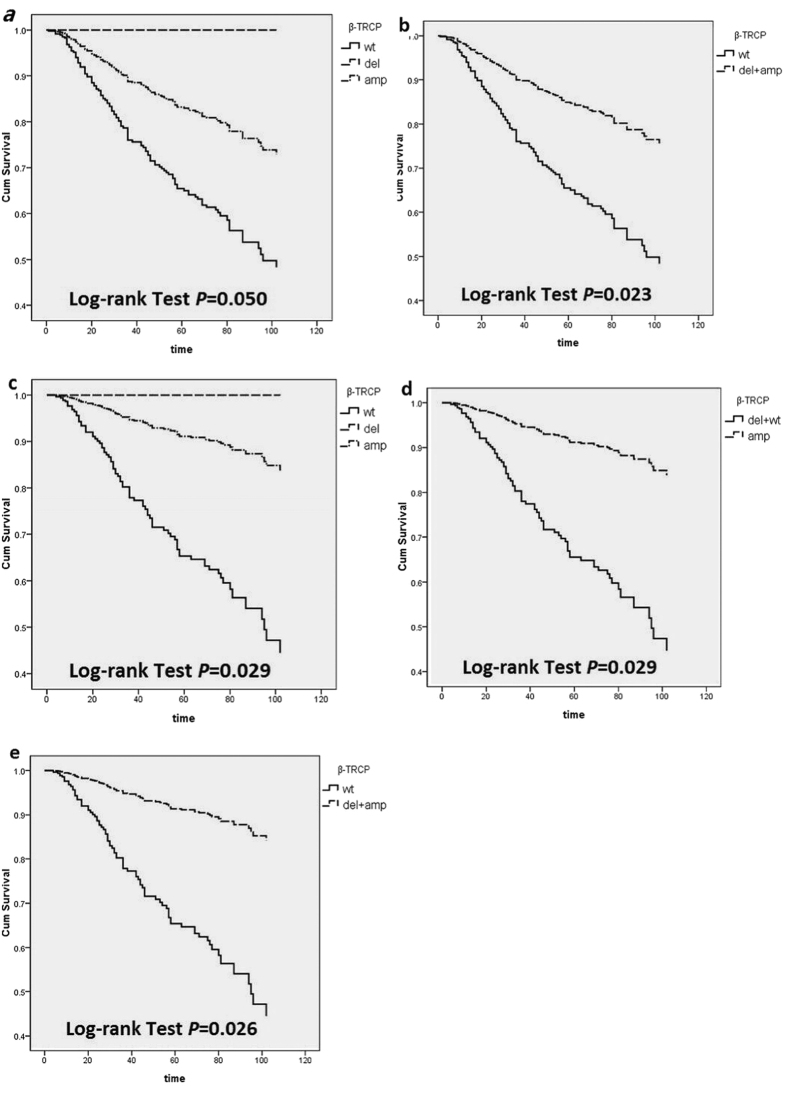
Kaplan–Meier curves of overall survival (OS) according to the five genes CNVs in patients with rectal cancer. (**a**) *β-TRCP* CNVs in CRC; (**b**) *β-TRCP* CNVs in combined model in CRC; (**c**) *β-TRCP* CNVs in rectal cancer; (**d**) *β-TRCP* amplification in rectal cancer; (**e**) *β-TRCP* CNVs in combined model in rectal cancer.

We find a marginally significant association between *β-TRCP* CNVs and CRC prognosis (amp *v.s*. wt, HR_adjusted_ = 0.42, 95% CI: 0.19, 0.97, *P* = 0.050). In the additive model, *β-TRCP* CNVs (del + amp) is significantly associated with CRC prognosis (del + amp *v.s.* wt, HR_adjusted_ = 0.39, 95% CI: 0.17, 0.88, *P* = 0.023) (Table 5, Fig. 1a,b). In the stratified analyses based on tumor location, the significant association between *β-TRCP* CNVs and CRC prognosis becomes marginally significant in rectal cancer (amp *v.s*. wt: HR_adjusted_ = 0.22, 95% CI: 0.06, 0.86, *P* = 0.029; amp *v.s*. del + wt,: HR_adjusted_ = 0.22, 95% CI: 0.06, 0.86, *P* = 0.029; del + amp *v.s.* wt: HR_adjusted_ = 0.21, 95% CI: 0.06, 0.83, *P* = 0.026), but not significant in colon cancer (Table 5, Fig. 1c–e). There was no statistically significant association between other gene CNVs and colon or rectal cancer in analyses stratified by tumor location (Table 5).

## Discussion

To our knowledge, this is the first study on the association between germline CNVs of *FBXW7, MDM2, SKP2, β-TRCP, NEDD4-1* and CRC risk and prognosis. In this study, *MDM2* CNVs significantly increase CRC risk, while *SKP2* CNVs significantly decrease CRC risk. We find evidence of three significant gene-environment interactions that increase risk of CRC: *SKP2* CNVs interact with consumption of fruit and fish consumption, and *FBXW7* CNVs interact with pork consumption. We also observe a significant association between *β-TRCP* CNVs and CRC prognosis.

We observe a significant association between *MDM2* amplification CNVs and CRC risk. However, there are few *MDM2* amplification among patients and controls (9 and 2 respectively), which limits statistical power. Because both amplification and deletion of MDM2 can increase CRC risk, the del + amp *v.s.* wt model can be viewed as a conservative estimate of the effect of *MDM2* on CRC risk. The amplification of *MDM2* may increase CRC risk by up to 14.40-fold, and the del + amp genotype of *MDM2* may also increase CRC risk by 6.35-fold. *MDM2* amplification was observed in 26 of 284 (9%) colorectal cancer tissue samples^33^, 14 of 80 (18%) CRCs tumor tissue samples^34^ and almost one-third of sarcomas^16^. *MDM2* could promote tumorigenesis by acting as a positive regulator of *p53* or independent of *p53*^35^. SNP data also indicate that even small differences in *MDM2* levels may affect cancer risk^36^. Moreover, *MDM2* also acts as a tumor suppressor through the Akt pathway, inducing the ubiquitination and degradation of *NFAT* (an invasion-promoting factor), thereby blocking cancer cell motility and invasion^37^. This could explain the significant association between *MDM2* CNVs and increased CRC risk in the del + amp *v.s.* wt model in our study. There is no significant association between *MDM2* CNVs and CRC prognosis. The role of *MDM2* in cancer prognosis is controversial, and may be affected by tumor variety and racial differences^31^,^38^,^39^.

We observed that *SKP2* CNVs (del + amp) are significantly associated with a 68% decreased risk of CRC. The overexpression of *SKP2* was associated with tumor differentiation, malignant transformation, and prognosis of malignant tumors^11^,^18^. *SKP2* gene amplification is commonly observed in metastatic tumors but not in early stage cancers^18^,^40^. Thus *SKP2* gene amplification is likely to be associated with advanced tumor progression. In our study, 60.9% of CRC patients were in stage I or II (Table 1). This may explain the non-significance of the association between *SKP2* CNVs and CRC risk. We did observe significant interactions between *SKP2* CNVs and fish or fruit consumption. Fish consumption has been reported to have protective effects in CRC^41^, which may be attributable to the omega-3 polyunsaturated fatty acids (PUFAs) in fish^42^. Omega-3s function as an anti-inflammatory, and is expected to have a function analogous to aspirin. Aspirin has been shown to reduce the incidence of CRC in both observational studies and randomized trials^43^,^44^. Dietary fiber in fruit is hypothesized to reduce the risk of CRC. Potential mechanisms for the protective effect dietary fibers include dilution of fecal carcinogens, reduction of transit time of feces through the bowel, and increased production of short chain fatty acids^45^,^46^,^47^.

*FBXW7* serves as a substrate adaptor for SCF ubiquitin ligase complex and mediates the recognition and binding of substrate proteins. SCF^*FBXW7*^ degrades several proteins with important roles in cell growth, proliferation, differentiation, and survival^48^. Previous studies have reported a tumor-suppressive function of *FBXW7* in colorectal tumor cells or tissues^23^,^30^, and copy number loss of *FBXW7* gene in tumor tissue was reported to be significantly associated with worse CRC prognosis^23^. The blood level of *FBXW7* expression has also been associated with the prognosis of breast cancer patients^49^. However, we did not observe any significant association between *FBXW7* CNVs and CRC risk or prognosis. The study by Chang *et al*. also found a non-significant association between *FBXW7* mRNA expression and CRC risk^10^, which is consistent with our results. About 6% of tumors harbor *FBXW7* loss-of-function variants, with different variants detected in different tumor types. This might reflect tissue-specific roles of *FBXW7* substrates^48^. A significant interaction effect has been observed between *FBXW7*, pork intake, and increased CRC risk. An updated meta-analysis of all prospective studies showed that the risk of CRC increased by 29% for every 100 g/d of red meat consumed^50^. The hard muscle fibers and high fat content in red meat may be the source of this association.

We found CRC patients with *β-TRCP* CNVs, have a better prognosis with a 58–61% OS increase. *β-TRCP* is the component of the ubiquitin ligase complex targeting *β-catenin* and *NF-ΚB* for proteasome degradation, which may contribute to the inhibition of apoptosis and to tumor metastasis^25^. Moreover, enhanced activity of *β-TRCP* has been widely observed in colorectal tumor cells and primary tumors^19^,^25^. The dual function of *β-TRCP* might explain the significant association between *β-TRCP* CNVs, and improved CRC prognosis. Different mechanisms of oncogenesis in rectal *vs*. colon cancer may explain why *β-TRCP* CNVs are only associated with rectal cancer prognosis in our study^51^. However we do not observe a significant association between *β-TRCP* CNVs and CRC risk. Mutations in *β-TRCP* are rarely detected in CRC, which is consistent with our results^52^,^53^.

Prior work has indicated that *NEDD4-1* may promote tumorigenesis by decreasing *PTEN* protein level, or through interference with the PI3K/AKT signaling pathway^54^,^55^. *NEDD4-1* is overexpressed in cancer cell lines^12^,^50^, animal models^56^,^57^,^58^, and in human cancer tissues^59^,^60^,^61^. However, we find no significant association between *NEDD4-1* CNVs and CRC risk or prognosis. Meanwhile, there have been no studies focused on the effect of *NEDD4-1* CNVs in peripheral blood on CRC risk and/or prognosis. One study indicated that SCF^*β-TRCP*^ can negatively regulate *NEDD4-1* stability, and *β-TRCP*-mediated destruction of the *NEDD4-1* oncoprotein may inhibit cell proliferation and migration^62^. This suggests that epistatic effects between *β-TRCP* and *NEDD4-1* may modify many signaling pathways. Further research is required to shed light on the relationship between these genes, and any differences that may exist between their functions in the germline *vs.* their function in tumor.

As in any case-control or prospective survival study, we must consider the limitations of our study. First, recall bias may be inevitable in the collection of data on environment factors. Second, we collected the frequency of soybean, sausage, fried food, and leftovers consumption without collecting information regarding quantity, which limits the statistical power of our analysis of gene-dietary interactions.

We find that *MDM2* and *SKP2* CNVs are significantly associated with CRC risk. In addition, we observe significant interaction effects between *SKP2* CNVs, fish or fruit consumption, and between *FBXW7* CNVs and pork intake, and CRC risk. There is a significant association between *β-TRCP* CNVs and CRC prognosis. Further research with larger sample sizes and more detailed functional evaluation will be required to confirm our results.

## Materials and Methods

### Subjects

After obtaining informed consent from study subjects, and approval from the Institutional Research Board of Harbin Medical University, we carried out the experiment in accordance with the relevant guidelines, including any relevant details. Informed consent was obtained from all subjects. We identified CRC patients who underwent surgery at the Cancer Hospital of Harbin Medical University, based on pathologic diagnosis without pre-selection. We excluded patients with neuroendocrine carcinoma, malignant melanoma, non-Hodgkin’s lymphoma, gastrointestinal stromal tumors, and Lynch syndrome CRC. From November 1^st^, 2004 to May 1^st^, 2010, we recruited 518 primary CRC patients. During the same period, we collected cancer free control subjects from the 2nd Affiliated Hospital of Harbin Medical University. We excluded controls with history of gastrointestinal disease according to self-report. 518 controls matched for age, gender, and residence were recruited.

### Data collection

We interviewed each participant face-to-face using a structured questionnaire with questions on demographic characteristics (age, gender, height and weight education and occupation), history of physical exercise, family history of cancer, and dietary status during the 12 months preceding the interview. We collected clinical information from medical records on tumor size, Dukes stage, chemotherapy treatment, histological and pathological types, and level of serum carcinoembryonic antigen (CEA) and carbohydrate antigen 19-9 (CA19-9). We followed up with 323 patients from November 2004 to March 2014. Overall survival (OS) was defined at the primary end point in our study. Survival time was calculated from the date of cancer diagnosis to death from colorectal cancer or other causes, or the time of follow-up. The date and cause of death of CRC patients were validated through the medical certification of death and the Harbin death registration system.

### DNA extraction and CNV detection

We extracted DNA from all 1036 blood samples (518 CRC and 518 controls) using QIAGEN DNeasy Blood & Tissue Kit. We detected *FBXW7, MDM2, SKP2, β-TRCP*, and *NEDD4-1* copy numbers using custom designed TaqMan Copy Number Assays (Supplementary Table S1). The quantitative assays were performed using the 7500 Fast Real-Time polymerase chain reaction machine in 96-well plates with a 10 ul reaction volume containing 20 ng DNA, 5 ul TaqMan Universal PCR Master Mix, 0.5 ul of the CNV assay, and 0.5 ul of the reference RNase P assay (Applied Biosystems, Carlsbad, Calif). The reaction was completed using the following cycling conditions: 95 °C for 15 seconds and 60 °C for 1 minute for 40 cycles. We used one sample with 2 copies of each CNV as a quality control in every 96-well assay plate (Supplementary Fig. 1). CNVs for each sample were detected three times. We analyzed data using 7500 software v2.0.6 (Applied Biosystems) to quantify the amplification cycle, and then imported the data to Copy Caller version 2.0 (Applied Biosystems) to estimate the gene copy numbers in every sample.

### Statistical analyses

We calculated the Hardy-Weinberg equilibrium in controls and compared using Fisher’s exact test. We evaluated homogeneity between cases and controls using Student’s *t-*test for continuous variables and a Chi-squared test for categorical variables. The unbalanced factors between the two groups were controlled for in a multivariable logistic regression for each gene, and in a multivariable logistic regression for gene-environment interactions. We used odds ratios (OR) and corresponding 95% confidence intervals (95% CI) to estimate the associations between *FBXW7, MDM2, SKP2, β-TRCP* and *NEDD4-1* CNVs and CRC risk via conditional logistic regression. We performed crossover analyses to evaluate gene-environment interaction effects on the risk of CRC with four types of OR (OR_e_, OR_g_, OR_eg_, OR_i_). We adjusted the heterogeneous demography characteristics in the conditional logistic regression. We defined 2 copies as the wild type (wt), more than 2 copies as the amplification type (amp) and less than 2 copies as the deletion type(del). Two additive models were applied in the conditional logistic regression analysis: amp *v.s.* del + wt and del + amp *v.s.* wt to estimate the association between CNVs CRC risk and prognosis. All statistical tests were two-sided, *P* value < 0.05 in the overall analysis. Adding a Bonferroni correction, a *P* value < 0.025 was used in stratified analyses. We used a multiple interpolation method to fill missing values in questionnaire responses (Supplementary Tables S4–S8). All statistical analyses were performed using SAS, version 9.2 (SAS Institute Inc.Cary, NC, USA).

## Additional Information

**How to cite this article**: Bi, H. *et al*. Copy number variation of E3 ubiquitin ligase genes in peripheral blood leukocyte and colorectal cancer. *Sci. Rep.* **6**, 29869; doi: 10.1038/srep29869 (2016).

## Supplementary Material

Supplementary Information

## Figures and Tables

**Table 1:** Basic characteristics of cases and controls.

**Characteristic**	**No. of Case (%)**	**No. of Controls (%)**	***P*****value**^**a**^
Age	60.45 ± 11.22	59.78 ± 10.64	0.955
Gender			0.002
Male	299 (57.7)	249 (48.1)	
Female	219 (42.3)	269 (51.9)	
BMI (kg/m^2^)	23.80 ± 3.80	24.10 ± 4.36	0.441
Education^**a**^			<0.001
Primary school and below	288 (58.6)	258 (51.3)	
Junior middle school	103 (21.0)	117 (23.3)	
Senior middle school and above	100 (20.4)	128 (25.4)	
Occupation^**a**^			<0.001
White collar	90 (17.9)	64 (12.8)	
Blue collar	260 (51.7)	319 (63.9)	
Both	153 (30.4)	116 (23.3)	
Family history of cancer^**a**^			<0.001
Yes	38 (10.2)	218 (43.1)	
No	334 (89.8)	288 (56.9)	
Location of primary tumor^**a**^			
Colon	262 (57.8)		
Rectum	191 (42.2)		
Stage of Dukes^**a**^			
I	52 (10.6)		
II	246 (50.3)		
III	159 (32.5)		
IV	32 (6.6)		
I+II	298 (60.9)		
III+IV	191 (39.1)		

^a^Missing data on subjects, education 27 cases, 15 controls; occupation, 15 cases, 19 controls; family history of cancer, 146 cases, 12 controls; tumor location, 65 cases.; stage of Dukes, 29 cases. ^b^*P* < 0.05 was considered statistically significant.

**Table 2:** Associations between CNVs and the risk of CRC.

**Gene**	**No. of Cases (%)**	**No. of Controls. (%)**	**Odds Ratio**^**a**^	**95% Confidence Interval**	***P*****Value**^**b**^
*MDM2*					
Wt	487 (96.0)	499 (98.4)	1.00		
Del	11 (2.2)	6 (1.2)	3.76	0.69, 20.61	0.127
Amp	9 (1.8)	2 (0.4)	**14.37**	**1.27, 163.74**	**0.032**
Amp *v.s.* del + wt			**14.40**	**1.26, 164.81**	**0.032**
Del + amp *v.s*. wt			**6.35**	**1.67, 24.19**	**0.007**
*SKP2*					
Wt	452 (95.4)	433 (91.4)	1.00		
Del	13 (2.7)	25 (5.2)	**0.32**	**0.10, 1.00**	**0.050**
Amp	9 (1.9)	16 (3.4)	0.32	0.10, 1.01	0.052
Amp *v.s.* del + wt			0.33	0.11, 1.02	0.055
Del + amp *v.s.* wt			**0.32**	**0.14, 0.72**	**0.006**
*FBXW7*					
Wt	410 (84.9)	400 (82.8)	1.00		
Del	37 (7.7)	36 (7.5)	1.29	0.61, 2.70	0.506
Amp	36 (4.4)	7 (9.7)	0.64	0.34, 1.23	0.181
Amp *v.s.* del + wt			0.63	0.33, 1.20	0.162
Del + amp *v.s.* wt			0.82	0.52, 1.42	0.557
*β-TRCP*					
Wt	465 (93.2)	469 (93.8)	1.00		
Del	5 (1.0)	8 (1.6)	0.71	0.11, 4.41	0.710
Amp	29 (5.8)	23 (4.6)	1.59	0.72, 3.52	0.252
Amp *v.s.* del + wt			1.59	0.72, 3.51	0.254
Del + amp *v.s.* wt			1.40	0.70, 2.88	0.363
*NEDD4-1*					
Wt	431 (89.0)	448 (92.6)	1.00		
Del	2 (0.4)	6 (1.2)	0.59	0.08, 4.55	0.614
Amp	51 (10.6)	30 (6.2)	1.44	0.73, 2.87	0.297
Amp *v.s.* del + wt			1.44	0.72, 2.87	0.297
Del + amp *v.s.* wt			1.31	0.39, 2.51	0.409

^a^Adjusted for gender, occupation, education, and family history of cancer. ^b^*P* < 0.05 in the conditional logistic regression analysis was considered statistically significant.

**Table 3:** Interactions between five gene CNVs and environmental factors on the risk of CRC.

**CNV genotypes**		**Environmental factors**		**Interaction**	
*SKP2*		Roughage (g/week)			
		<50	≥50		
		OR_eg_ (95% CI)^a^		OR_i_ (95% CI)^a^	*P* value^b^
	Del + wt	1.00	0.58 (0.39, 0.88)		
	Amp	0.18 (0.03, 0.98)	**0.18 (0.03, 0.99)**	1.72 (0.16, 18.74)	0.657
	Wt	1.00	0.62 (0.41, 0.94)		
	Del + amp	0.28 (0.08, 1.00)	**0.13 (0.04, 0.44)**	0.74 (0.13, 4.30)	0.734
		Fruit (times/week)			
		<2	≥2		
		OR_eg_ (95% CI)^a^		OR_i_ (95% CI)^a^	*P* value^b^
	Del + wt	1.00	0.61 (0.40, 0.94)		
	Amp	0.82 (0.03, 0.53)	0.70 (0.15, 3.22)	13.89 (1.20, 160.57)	0.035
	Wt	1.00	0.62 (0.34, 0.98)		
	Del + amp	0.09 (0.02, 0.44)	**0.33 (0.12, 0.96)**	6.10 (0.92, 40.38)	0.061
		Fish (times/week)			
		≤1	>1		
		OR_eg_ (95% CI)^a^		OR_i_ (95% CI)^a^	*P* value^b^
	Wt	1.00	0.30 (0.17, 0.52)		
	Del + amp	0.09 (0.03, 0.31)	0.39 (0.08, 2.00)	13.62 (1.70, 109.36)	**0.014**
*MDM2*		Refined grains (g/day)			
		≤250	>250		
		OR_eg_ (95% CI)^a^		OR_i_ (95% CI)^a^	*P* value^b^
	Wt	1.00	2.53 (1.59, 4.02)		
	Del + amp	13.35 (2.13, 89.49)	**5.44 (1.03, 28.86)**	0.09 (0.00, 2.14)	0.135
		Fat meat			
		No	Yes		
		OR_eg_ (95% CI)^a^		OR_i_ (95% CI)^a^	*P* value^b^
	Wt	1.00	2.30 (1.48, 3.57)		
	Del + amp	11.25 (1.63, 77.25)	**8.55 (1.22, 59.75)**	0.30 (0.02, 5.09)	0.427
		Egg (/week)			
		≤3	>3		
		OR_eg_ (95% CI)^a^		OR_i_ (95% CI)^a^	*P* value^b^
	Wt	1.00	1.60 (1.08, 2.37)		
	Del + amp	9.31 (0.66, 131.06)	**7.33 (1.57, 34.30)**	0.49 (0.02, 10.06)	0.645
		Chinese pickled sour cabbage (times/month)			
		≤2	>2		
		OR_eg_ (95% CI)^a^		OR_i_ (95% CI)^a^	*P* value^b^
	Wt	1.00	2.05 (1.35, 3.12)		
	Del + amp	6.41 (1.16, 35.27)	**27.61 (2.12, 259.81)**	2.10 (0.10, 44.07)	0.633
		leftovers (times/week)^c^			
		≤3	>3		
		OR_eg_ (95% CI)^a^		OR_i_ (95% CI)^a^	*P* value^b^
	Wt	1.00	1.873 (1.234, 2.843)		
	Del + amp	3.30 (0.37, 29.54)	**26.67 (2.62, 271.60)**	4.31 (0.18, 100.81)	0.363
*FBXW7*		Roughage (g/week)			
		<50	≥50		
		OR_eg_ (95% CI)^a^		ORi (95% CI)^a^	*P* value^b^
	Del + wt	1.00	0.62 (0.41, 0.95)		
	Amp	0.57 (0.22, 1.50)	**0.37 (0.15, 0.91)**	1.04 (0.30, 3.67)	0.946
		Fish (times/week)			
		≤1	>1		
		OR_eg_ (95% CI)^a^		ORi (95% CI)^a^	*P* value^b^
	Del + wt	1.00	0.34 (0.24, 0.66)		
	Amp	0.64 (0.28, 1.44)	**0.25 (0.07, 0.94)**	0.99 (0.20, 4.88)	0.993
		Refined grains (g/day)			
		≤250	>250		
		OR_eg_ (95% CI)^a^		OR_i_ (95% CI)^a^	*P* value^b^
	Wt	1.00	2.28 (1.37, 3.80)		
	Del + amp	0.87 (0.45, 1.67)	****2.81 (1.52, 6.86)****	1.43 (0.49, 4.19)	0.519
		Fat meat			
		No	Yes		
		OR_eg_ (95% CI)^a^		OR_i_ (95% CI)^a^	*P* value^b^
	Wt	1.00	2.23 (1.35, 3.68)		
	Del + amp	0.76 (0.40, 1.47)	**2.30 (1.03, 5.11)**	1.35 (0.47, 3.82)	0.577
		Pork (g/week)			
		≤250	>250		
		OR_eg_ (95% CI)^a^		OR_i_ (95% CI)^a^	*P* value^b^
	Wt	1.00	1.34 (0.87, 2.06)		
	Del + amp	0.46 (0.21, 0.99)	1.92 (0.90, 4.11)	**3.13 (1.06, 9.41)**	**0.040**
		Physical exercise			
		No	Yes		
		OR_eg_ (95% CI)^a^		OR_i_ (95% CI)^a^	*P* value^b^
	Wt	1.00	0.06 (0.02, 0.20)		
	Del + amp	1.58 (0.66, 3.78)	**0.06 (0.01, 0.31)**	0.65 (0.10, 4.47)	0.662
*NEDD4-1*		Refined grains (g/day)			
		≤250	>250	interaction	
		OR_eg_ (95% CI)^a^		OR_i_ (95% CI)^a^	*P* value^b^
	Del + wt	1.00	2.61 (1.61, 4.24)		
	Amp	1.63 (0.56, 4.71)	**2.83 (1.02, 7.88)**	0.67 (0.15, 2.89)	0.587
	Wt	1.00	2.63 (1.62, 4.27)		
	Del + amp	1.48 (0.57,3.86)	**2.75 (1.00, 7.55)**	0.71 (0.18, 2.81)	0.622
		Chinese pickled sour cabbage (times/month)			
		≤2	>2		
		OR_eg_ (95% CI)^1^		OR_i_ (95% CI)^a^	*P* value^b^
	Del + wt	1.00	1.86 (1.21, 2.85)		
	Amp	3.13 (0.92, 10.62)	**3.59 (1.23, 10.48)**	1.79 (0.41, 7.88)	0.444
		Fat meat			
		No	Yes		
		OR_eg_ (95% CI)^a^		OR_i_ (95% CI)^a^	*P* value^b^
	Del + wt	1.00	2.26 (1.43, 3.58)		
	Amp	1.03 (0.35, 3.00)	**3.60 (1.27, 10.19)**	1.54 (0.35, 6.71)	0.564
	Wt	1.00	2.27 (1.43, 3.60)		
	Del + amp	1.00 (0.38, 2.62)	**3.30 (1.21, 9.14)**	1.46 (0.37, 5.84)	0.590
		Physical exercise			
		No	Yes		
		OR_eg_ (95% CI)^a^		OR_i_ (95% CI)^a^	*P* value^b^
	Wt	1.00	0.06 (0.02, 0.20)		
	Del + amp	1.58 (0.66, 3.78)	**0.06 (0.01, 0.31)**	10.27 (0.60, 177.48)	0.109

^a^Adjusted for gender, occupation, education, and family history of cancer. ^b^*P* < 0.05 in the conditional logistic regression analysis was considered statistically significant. ^c^ leftovers: leftovers more than 12 hours.

**Table 4:** Clinical and pathological features of 323 CRC patients.

**Characteristics**	**Patients**	**%**
Age at diagnosis		
<50	74	22.9
50–60	112	34.7
60–70	91	28.2
>70	46	14.2
Mean	58.58 ± 10.68	
Median survival time (month)	73	
Extreme value	0–109	
Gender		
Male	182	56.3
Female	141	43.7
Location of primary tumer^a^		
Colon	108	33.5
Rectum	214	66.5
CEA level (ng/ul)		
<5	141	43.7
≥5	182	56.3
CA19-9 level (U/ml)^a^		
<37	240	74.8
≥37	81	25.2
Pathological type^a^		
Protrude type	202	64.7
Infiltrating or ulcerative type	107	34.3
Others	3	1.0
Anastomat on surgery^a^		
Yes	228	71.9
No	75	23.7
Unknown	14	4.4
Stage of Dukes^a^		
I	39	12.1
II	142	44.1
III	119	37.0
IV	22	6.8
I+II	181	56.2
III+IV	141	43.8
Histological type		
Adenocarcinoma	249	77.1
Mucinous adenocarcinoma	63	19.5
Other types	11	3.4
Degree of differentiation		
Low	49	15.2
Medium	258	79.9
High	3	0.9
Unknown	13	4.0
Chemotherapy treatment^a^		
No	186	57.9
FOLFOX4	45	14.0
XELOX	22	6.9
LCF	44	13,7
5-Fu	6	1.9
Others	18	5.6
Metastasis		
Yes	141	43.7
No	182	46.3
Prognosis		
Death	136	42.1
living	146	45.2
Losing follow-up	41	12.7

CA19-9, carbohydrate antigen19-9; CEA, carcinoembryonic antigen; CI, confidence interval. ^a^Missing data on subjects, tumor location, one case; CA19-9 level, two cases; pathological type, 11 cases; anastomat on surgery, six cases; stage of Dukes, 22 cases; chemotherapy treatment, 2 cases.

**Table 5:** The relationships between gene CNVs and prognosis of CRC.

**CNV Genotypes**	**Total patients (n = 323)**					**P Value**^**b**^	**Colon cancer (n = 108)**					**P Value**^**c**^	**Rectal cancer (n = 214)**					**P Value**^**c**^
	**Patients (%)**	**5-Year Survival (%)**	**3-Year Survival (%)**	**OS (Mean (SD)) (month)**	**HR (95% CI)**^**a**^		**Patients (%)**	**5-year survival (%)**	**3-year survival (%)**	**OS (Mean (SD)) (month)**	**HR (95% CI)**^**a**^		**Patients (%)**	**5-year survival (%)**	**3-year survival (%)**	**OS (Mean (SD)) (month)**	**HR (95% CI)**^**a**^	
*FBXW7*																		
Wt	255 (83.6)	59	68	74.00 (2.53)	1.00		88 (85.4)	62	71	77.32 (4.35)	1.00		166 (82.5)	58	68	72.48 (3.09)	1	
Del	27 (8.9)	68	76	82.75 (7.61)	0.62 (0.29, 1.33)	0.220	10 (9.7)	48	70	68.95 (11.96)	1.09 (0.41, 2.88)	0.868	17 (8.5)	81	81	90.74 (9.01)	0.31 (0.07, 1.26)	0.100
Amp	23 (7.5)	61	70	79.35 (8.18)	0.91 (0.46, 1.83)	0.793	5 (4.9)	20	40	35.40 (12.23)	3.39 (1.03, 11.18)	0.045	18 (9.0)	72	78	89.61 (7.71)	0.48 (0.18, 1.29)	0.146
Amp *v.s.* del + wt					0.95 (0.48, 1.90)	0.888					3.38 (1.05, 11.12)	0.045					0.51 (0.19, 1.38)	0.185
Del + amp *v.s.* wt					0.75 (0.44, 1.29)	0.299					1.57 (0.74, 3.34)	0.242					0.41 (0.18, 0.92)	0.031
*MDM2*																		
Wt	298 (94.6)	60	68	74.28 (2.35)	1.00		99 (96.1)	56	68	73.5 (4.18)	1.00		198 (93.8)	60	69	74.97 (2.84)	1	
Del	9 (2.9)	67	67	75.22 (14.09)	0.72 (0.23, 2.29)	0.580	4 (3.9)	75	75	81.25 (20.59)	0.62 (0.08, 4.61)	0.640	5 (2.4)	60	60	55.40 (13.56)	1.13 (0.27, 4.80)	0.867
Amp	8 (2.5)	61	61	60.60 (9.15)	0.80 (0.25, 2.57)	0.707	0						8 (3.8)	61	61	60.60 (9.15)	0.96 (0.30, 3.13)	0.956
Amp *v.s.* del + wt					0.81 (0.25, 2.61)	0.727											0.96 (0.30, 3.11)	0.949
Del + amp *v.s.* wt					0.76 (0.33, 1.75)	0.517					0.62 (0.08, 4.61)	0.640					1.03 (0.40, 2.61)	0.957
*SKP2*																		
Wt	284 (95.3)	60	70	75.34 (2.39)	1.00		95 (95.0)	58	69	74.9 (44.23)	1.00		188 (95.5)	61	70	75.79 (2.90)	1	
Del	6 (2.0)	50	67	68.50 (15.37)	1.31 (0.41, 4.17)	0.658	3 (3.0)	33	67	43.33 (16.85)	3.62 (0.80, 16.31)	0.094	3 (1.5)	67	67	84.00 (15.51)	0.75 (0.10, 5.55)	0.777
Amp	8 (2.7)	42	42	50.03 (11.55)	1.39 (0.51, 3.79)	0.526	2 (2.0)			19.50 (3.89)	4.68 (0.57, 38.39)	0.151	6 (3.0)	46	46	53.83 (12.53)	0.99 (0.30, 3.22)	0.985
Amp *v.s.* del + wt					1.38 (0.50, 3.78)	0.531					4.47 (0.55, 36.48)	0.162					0.99 (0.31, 3.22)	0.990
Del + amp *v.s.* wt					1.35 (0.62, 2.91)	0.447					3.92 (1.13, 3.66)	0.032					0.93 (0.33, 2.54)	0.865
*β-TRCP*																		
Wt	282 (91.0)	58	68	74.09 (2.42)	1.00		90 (89.1)	58	68	73.97 (4.41)	1.00		191 (91.8)	59	68	74.47 (2.89)	1	
Del	4 (1.3)	100	100	90.53 (11.69)		0.962	3 (3.0)	100	100	86.00 (14.69)		0.976	1 (0.5)	100	100	79		0.971
Amp	24 (7.7)	66	74	63.25 (5.44)	**0.42 (0.19, 0.97)**	**0.050**	8 (7.9)	50	75	61.00 (8.08)	0.61 (0.18, 2.03)	0.421	16 (7.7)	74	74	64.01 (7.04)	**0.22 (0.06, 0.86)**	**0.029**
Amp *v.s.* del + wt					0.44 (0.19, 1.01)	0.052					0.63 (0.19, 2.08)	0.444					**0.22 (0.06, 0.86)**	**0.029**
Del + amp *v.s.* wt					**0.39 (0.17, 0.88)**	**0.023**					0.50 (0.16, 1.57)	0.230					**0.21 (0.06, 0.83)**	**0.026**
*NEDD4-1*																		
Wt	269 (89.7)	59	68	74.39 (2.46)	1.00		89 (87.9)	56	68	73.35 (4.44)	1.00		181 (90.5)	60	68.4	75.23 (2.94)	1	
Del	2 (0.6)	50	50	46.00 (23.34)	1.02 (0.14, 7.66)	0.985	0						2 (1.0)	50	50	46.00 (23.34)	0.85 (0.11, 6.81)	0.878
Amp	29 (9.7)	65	72	74.79 (7.61)	0.82 (0.40, 1.70)	0.597	12 (12.1)	58	67	70.83 (12.27)	1.06 (0.41, 2.76)	0.900	17 (8.5)	77	76	70.12 (8.26)	0.73 (0.23, 2.35)	0.595
Amp *v.s.* del + wt					0.82 (0.40, 1.70)	0.597					1.06 (0.41, 2.76)	0.900					0.73 (0.23, 2.35)	0.595
Del + amp *v.s.* wt					0.84 (0.42, 1.67)	0.620					1.06 (0.41, 2.76)	0.900					0.75 (0.27, 2.09)	0.578

CI, confidence interval; HR, hazard ratio; OS, overall survival.

^a^adjusted for CEA and CA19-9 level before surgery, Dukes stage, pathological type and metastasis. ^b^*P* < 0.05 in the survival analysis was considered statistically significant. ^c^*P* < 0.025 in the stratified survival analysis by location was considered statistically significant.
