# Supplementary material for: Copy number variation of E3 ubiquitin ligase genes in peripheral blood leukocyte and colorectal cancer
Source: Sci Rep. 2016 Jul 15;6:29869. doi: 10.1038/srep29869 (PMC4945909; doi:10.1038/srep29869)

Copy number variation of E3 ubiquitin ligase genes in peripheral blood leukocyte and colorectal cancer

Haoran Bi[1], Tian Tian[1], Lin Zhu[1], Haibo Zhou[1], Hanqing Hu[2], Yanhong Liu[3], Xia Li[4], Fulan Hu[1*], Yashuang Zhao[1*], Guiyu Wang[2*]

Author Affiliations: [1]Department of Epidemiology, Public Health College, Harbin Medical University, 157 Baojian Street, Harbin, Heilongjiang, People's Republic of China. [2]Department of Colorectal Cancer Surgery, The Second Affiliated Hospital of Harbin Medical University, 246 Xuefu Street, Harbin, Heilongjiang, People's Republic of China. [3]Department of Clinical Laboratory, The Second Affiliated Hospital of Harbin Medical University, 246 Xuefu Street, Harbin, Heilongjiang, People's Republic of China. [4]College of Bioinformatics Science and Technology, Harbin Medical University, 157 Baojian Street, Harbin, Heilongjiang, People's Republic of China.

Supplementary Table S1. The pater number of customed TaqMan copy number assays.

FBXW7 assay	ID#hs02920964_cn
MDM2 assay	ID#hs03082319_cn
SKP2 assay	ID#hs06086556_cn
β-TRCP assay	ID#hs03734483_cn
NEDD4-1 assay	ID#hs02225249_cn

Supplementary Table S2. Copy number variations of matched case-control pairs.

FBXW7[a]		**Case No.**		
		Wt	Del	Amp
**Control No.**	Wt	336	33	30
	Del	32	2	2
	Amp	41	2	4
MDM2[a]		**Case No.**		
		Wt	Del	Amp
**Control No.**	Wt	480	10	9
	Del	6	0	0
	Amp	1	1	0
SKP2[a]		**Case No.**		
		Wt	Del	Amp
**Control No.**	Wt	411	12	8
	Del	39	1	1
	Amp	0	0	0

β-TRCP[a]		Case No.		
		Wt	Del	Amp
Control No.	Wt	433	5	28
	Del	8	0	0
	Amp	22	0	1

NEDD4-1[a]		Case No.		
		Wt	Del	Amp
Control No.	Wt	396	2	49
	Del	6	0	0
	Amp	28	0	2

[a] missing data, for *FBXW7* 70 values; for *MDM2* 22 values; for *SKP2* 88 values; for *β-TRCP* 37 values; for *NEDD4-1*, 68 values.

Supplementary Table S3. Association of environment factors and CRC risk.

Environmental Factors	OR[a] (95%CI)	*P* Value[b]
Refined grains (g/day)		
≤ 250	1.00	
> 250	2.36 (1.50, 3.70)	**<0.001**
Roughage (g/week)		
< 50	1.00	
≥ 50	0.66 (0.45, 0.96)	**0.025**
Vegetable (times/week)		
< 2	1.00	
≥ 2	0.94 (0.63, 1.39)	0.755

Fruit (times/week)		
< 2	1.00	
≥ 2	0.64 (0.42, 0.95)	**0.029**
Fat meat		
No	1.00	
Yes	2.13 (1.40, 3.24)	**<0.001**
Beef (g/week)		
< 250	1.00	
≥ 250	0.93 (0.62, 1.39)	0.705
Pork (g/week)		
< 250	1.00	
≥ 250	1.61 (1.09, 2.37)	**0.016**
Poultry (g/week)		
< 250	1.00	
≥ 250	1.30 (0.87, 2.00)	0.192
Fish (times/week)		
≤ 1	1.00	
> 1	0.43 (0.27, 0.68)	**<0.001**
Seafood (times/week)		
≤ 1	1.00	
> 1	0.75 (0.47, 1.25)	0.271
Braised fish (times/week)		
≤ 1	1.00	
> 1	1.18 (0.79, 1.78)	0.419
Bean products (times/week)		
≤ 1	1.00	

> 1	1.19 (0.77, 1.86)	0.427
Milk (times/week)		
≤ 2	1.00	
> 2	1.09 (0.68, 1.75)	0.715
Egg (/week)		
≤ 3	1.00	
> 3	1.67 (1.14, 2.45)	**0.008**
Coffee		
yes	1.00	
no	0.75 (0.26, 2.18)	0.595
Tea		
yes	1.00	
no	0.65 (0.40, 1.05)	0.075
Fried food (times/month)		
≤ 1	1.00	
> 1	1.57 (0.98, 2.53)	**0.062**
Sausage (times/month)		
≤ 1	1.00	
> 1	3.12 (1.55, 6.28)	**0.001**
Spicy food (times/week)		
≤ 3	1.00	
> 3	0.81 (0.55, 1.19)	0.283
Chinese pickled sour cabbage (times/month)		
≤ 2	1.00	
> 2	1.98 (1.33, 2.94)	**0.001**
Leftovers [c] (times/week)		

≤ 3	1.00	
> 3	1.90 (6.27, 2.85)	**0.002**
Canned fruit (times/week)		
≤ 3	1.00	
> 3	1.36 (0.66, 2.82)	0.41
Canned meat (times/week)		
≤ 3	1.00	
> 3	1.04 (0.41, 2.64)	0.941
Physical exercise		
No	1.00	
Yes	0.05 (0.02, 0.15)	**<0.001**

[a] adjusted for gender, occupation, education, and family history of cancer. [b] $P < 0.05$ in the conditional logistic regression analysis was considered statistically significant. [c] leftovers: leftovers more than 12 hours

Supplementary Table S4. Associations between CNVs and the risk of CRC after interpolation.

Gene	No. of Cases (%)	No. of Controls. (%)	Odds Ratio[a] (95% Confidence Interval)	*P* Value[b]
MDM2				
Wt	487 (96.0)	499 (98.4)	1.00	
Del	11 (2.2)	6 (1.2)	2.38 (0.65,8.77)	0.191
Amp	9 (1.8)	2 (0.4)	**15.44 (2.05,116.50)**	**0.008**
Amp *v.s.* del+wt			**12.55 (1.74, 90.53)**	**0.012**
Del+amp *v.s.* wt			**4.13 (1.36, 12.11)**	**0.013**

SKP2				
Wt	452 (95.4)	433 (91.4)	1.00	
Del	13 (2.7)	25 (5.2)	0.52 (0.24, 1.11)	0.091
Amp	9 (1.9)	16 (3.4)	0.45 (0.17, 1.17)	0.100
Amp *v.s.* del+wt			0.46 (0.18, 1.19)	0.109
Del+amp *v.s.* wt			**0.49 (0.27, 0.89)**	**0.020**
FBXW7				
Wt	410 (84.9)	400 (82.8)	1.00	
Del	37 (7.7)	36 (7.5)	1.41 (0.79, 2.53)	0.245
Amp	36 (4.4)	47 (9.7)	0.71 (0.42, 1.22)	0.216
Amp *v.s.* del+wt			0.70 (0.41, 1.19)	0.185
Del+amp *v.s.* wt			0.97 (0.65, 1.45)	0.883
β-TRCP				
Wt	465 (93.2)	469 (93.8)	1.00	
Del	5 (1.0)	8 (1.6)	0.42 (0.10, 1.76)	0.232
Amp	29 (5.8)	23 (4.6)	1.71 (0.88, 3.32)	0.117
Amp *v.s.* del+wt			1.70 (0.87, 3.31)	0.118
Del+amp *v.s.* wt			1.31 (0.72, 2.39)	0.383
NEDD4-1				
Wt	431 (89.0)	448 (92.6)	1.00	
Del	2 (0.4)	6 (1.2)	0.43 (0.07, 2.60)	0.358
Amp	51 (10.6)	30 (6.2)	1.65 (0.92, 2.95)	0.092
Amp *v.s.* del+wt			1.65 (0.92, 2.94)	0.093
Del+amp *v.s.* wt			1.46 (0.84, 2.48)	0.183

[a] adjusted for gender, occupation, education, and family history of cancer. [b] $P < 0.05$ in the conditional logistic regression analysis was considered statistically significant.

Supplementary Table S5. Interactions Between Five Gene amplifications and Environmental Factors on the Risk of CRC.

Environmental Factors	*MDM2*		Interaction		*SKP2*		Interaction	
	Del+wt	Amp			Del+wt	Amp		
		OR_{eg} (95% CI)[a]	OR_i (95% CI)[a]	*P* Value[b]		OR_{eg} (95% CI)[a]	OR_i (95% CI)[a]	*P* Value[b]
Refined grains (g/day)								
≤ 250	1.00	14.80 (1.22, 179.50)		0.941	1.00	0.21 (0.04, 1.18)	1.40 (0.09, 20.79)	0.807
> 250	2.38 (1.52, 3.75)				2.48 (1.52, 4.03)	0.73 (0.09, 5.98)		
Roughage (g/week)								
< 50	1.00			0.800	1.00	0.18 (0.03, 0.98)	1.72 (0.16, 18.74)	0.657
≥ 50	0.66 (0.45, 0.96)	4.54 (0.25, 82.99)			0.58 (0.39, 0.88)	**0.18 (0.03, 0.99)**		
Fruit (times/week)								
< 2	1.00			0.866	1.00	0.82 (0.03, 0.53)	**13.89 (1.20, 160.57)**	**0.035**
≥ 2	0.62 (0.41, 0.93)	9.41 (0.53, 167.21)			0.61 (0.40, 0.94)	0.70 (0.15, 3.22)		
Fat meat								
No	1.00	19.09 (1.26, 288.12)		0.871	1.00	0.62 (0.15, 2.54)	0.16 (0.01, 2.55)	0.195
Yes	2.30 (1.49, 3.56)				2.52 (1.59, 3.99)	0.25 (0.02, 2.59)		
Pork (g/week)								
< 250	1.00			0.803	1.00	0.29 (0.04, 2.36)	1.06 (0.07, 16.23)	0.964
≥ 250	1.67 (1.21, 2.48)	8.42 (0.36, 197.09)			1.56 (1.04, 2.34)	0.48 (0.08, 2.84)		
Fish (times/week)								
≤ 1	1.00			0.847	1.00	0.21 (0.05, 0.90)	2.14 (0.09, 52.10)	0.642
> 1	0.39 (0.24, 0.64)	7.67 (0.41, 142.10)			0.39 (0.24, 0.65)	0.18 (0.01, 2.92)		
Egg (/week)								

≤ 3	1.00			0.823	1.00	0.21 (0.02, 1.96)	1.82 (0.13, 27.05)	0.653
> 3	1.66 (1.12, 2.44)	11.31 (0.85, 151.23)			1.78 (1.18, 2.68)	0.69 (0.16, 3.04)		
Fried food (times/month)								
≤ 1	1.00			0.819	1.00	0.30 (0.09, 1.00)	2.13 (0.04, 112.23)	0.709
> 1	1.50 (0.92, 2.44)	10.04 (0.56, 179.02)			1.46 (0.88, 2.41)	0.93 (0.02, 40.64)		
Sausage (times/month)								
≤ 1	1.00		1.92 (0.26, 14.12)	0.525	1.00			0.821
> 1					3.84 (1.73, 8.50)	2.51 (0.01, 734.76)		
Chinese pickled sour cabbage (times/month)								
≤ 2	1.00			0.805	1.00	0.11 (0.02, 0.69)	7.77 (0.67, 89.60)	0.100
> 2	2.11 (1.39, 3.19)	17.17 (0.86, 343.75)			1.91 (1.25, 2.94)	1.65 (0.32, 8.48)		
Leftovers [c] (times/week)								
≤ 3	1.00	4.58 (0.11, 188.55)		0.743	1.00	0.35 (0.09, 1.39)	1.21 (0.09, 16.72)	0.889
> 3	1.86 (1.23, 2.82)				2.07 (1.34, 3.18)	0.88 (0.10, 8.33)		
Physical exercise								
No	1.00	12.25 (0.48, 310.35)		0.816	1.00	0.24 (0.05, 1.30)		0.926
Yes	0.04 (0.01, 0.13)				0.05 (0.02, 0.17)			

Continued.

**Environmental Factors**	***FBXW7***		**Interaction**		***NEDD4-1***		**Interaction**	
	**Del+wt**	**Amp**			**Del+wt**	**Amp**		
	**OR_{eg} (95% CI)[a]**		**OR_i (95% CI)[a]**	***P* Value[b]**	**OR_{eg} (95% CI)[a]**		**OR_i (95% CI)[a]**	***P* Value[b]**
Refined grains (g/day)								
≤ 250	1.00	0.44 (0.18, 1.05)	2.70 (0.65, 11.26)	0.174	1.00	1.63 (0.56, 4.71)	0.67 (0.15, 2.89)	0.587
> 250	2.18 (1.33, 3.57)	2.57 (0.84, 7.85)			2.61 (1.61, 4.24)	**2.83 (1.02, 7.88)**		
Roughage (g/week)								

< 50	1.00	0.57 (0.22, 1.50)	1.05 (0.30, 3.67)	0.946	1.00	0.88 (0.59, 1.31)	1.20 (0.25, 5.77)	0.823
≥ 50	0.62 (0.41, 0.95)	**0.37 (0.15, 0.91)**			1.30 (0.35, 4.81)	1.36 (0.59, 3.13)		
Fruit (times/week)								
< 2	1.00	0.39 (0.14, 1.10)	2.92 (0.72, 11.73)	0.132	1.00	0.94 (0.33, 2.70)	1.51 (0.33, 6.92)	0.595
≥ 2	0.59 (0.38, 0.91)	0.67 (0.28, 1.62)			0.60 (0.39, 0.93)	0.86 (0.28, 2.59)		
Fat meat								
No	1.00	0.43 (0.17, 1.08)	2.43 (0.62, 9.49)	0.204	1.00	1.03 (0.35, 3.00)	1.54 (0.35, 6.71)	0.546
Yes	2.16 (1.35, 3.47)	2.27 (0.80, 6.45)			2.26 (1.43, 3.58)	**3.60 (1.27, 10.19)**		
Pork (g/week)								
< 250	1.00	0.37 (0.14, 0.98)	2.83 (0.76, 10.59)	0.123	1.00	2.43 (0.87, 6.80)	0.46 (0.09, 2.06)	0.289
≥ 250	1.46 (0.96, 2.21)	1.53 (0.60, 3.87)			1.71 (1.13, 2.59)	1.77 (0.57, 5.51)		
Fish (times/week)								
≤ 1	1.00	0.64 (0.28, 1.44)	0.99 (0.20, 4.88)	0.993	1.00	1.02 (0.44, 2.38)	3.00 (0.54, 17.72)	0.224
> 1	0.34 (0.24, 0.66)	**0.25 (0.07, 0.94)**			0.39 (0.24, 0.64)	1.20 (0.27, 5.39)		
Egg (/week)								
≤ 3	1.00	0.64 (0.26, 1.66)	1.17 (0.31, 4.40)	0.812	1.00	3.53 (1.14, 10.88)	0.23 (0.05, 1.06)	0.059
> 3	1.59 (1.05, 2.42)	1.20 (0.46, 3.09)			1.88 (1.24, 2.85)	1.52 (0.57, 4.09)		
Fried food (times/month)								
≤ 1	1.00	0.43 (0.20, 0.93)	4.38 (0.94, 20.40)	0.060	1.00	1.47 (0.65, 3.31)	1.07 (0.20, 5.78)	0.938
> 1	1.22 (0.73, 2.05)	2.29 (0.63, 8.34)			1.48 (0.89, 2.48)	2.32 (0.55, 9.90)		
Sausage (times/month)								
≤ 1	1.00	0.88 (0.29, 2.71)	0.32 (0.04, 2.59)	0.286	1.00	3.13 (0.92, 10.62)		0.833
> 1	4.11 (1.79, 9.48)	1.16 (0.21, 6.60)			3.06 (1.45, 6.47)			
Chinese pickled sour cabbage (times/month)								
≤ 2	1.00	0.93 (0.39, 2.23)	0.51 (0.14, 1.83)	0.296	1.00	1.08 (0.42, 2.80)	1.79 (0.41, 7.88)	0.444
> 2	2.10 (1.37, 3.22)	0.98 (0.36, 2.64)			1.86 (1.21, 2.85)	**3.59 (1.23, 10.48)**		

Leftovers [c] (times/week)								
≤ 3	1.00	0.69 (0.29, 1.63)	0.74 (0.20, 2.74)	0.296	1.00	1.74 (0.70, 4.27)	0.76 (0.17, 3.46)	0.720
> 3	2.02 (1.30, 3.13)	1.04 (0.40, 2.70)			1.96 (1.28, 3.02)	2.59 (0.77, 8.64)		
Physical exercise								
No	1.00	1.74 (0.56, 5.34)		0.840	1.00	1.20 (0.43, 3.36)	8.51 (0.48, 152.45)	0.146
Yes	0.07 (0.02, 0.21)				0.03 (0.01, 0.14)	0.33 (0.03, 3.09)		

Continued.

**Environmental Factors**	***β-TRCP***		**Interaction**	
	**Del+wt**	**Amp**		
	**OR_{eg} (95% CI)[a]**		**OR_i (95% CI)[a]**	***P* Value[b]**
Refined grains (g/day)				
≤ 250	1.00	1.98 (0.62, 6.29)	0.63 (0.11, 3.52)	0.601
> 250	2.57 (1.59, 4.15)	3.22 (0.91, 11.40)		
Roughage (g/week)				
< 50	1.00	0.75 (0.22, 2.56)	3.91 (0.74, 20.70)	0.108
≥ 50	0.56 (0.38, 0.84)	1.65 (0.54, 5.03)		
Fruit (times/week)				
< 2	1.00	2.27 (0.63, 8.21)	0.60 (0.11, 3.23)	0.555
≥ 2	0.62 (0.40, 0.97)	0.85 (0.30, 2.40)		
Fat meat				
No	1.00	2.52 (0.79, 8.05)	0.31 (0.05, 1.80)	0.192
Yes	2.28 (1.47, 3.53)	1.79 (0.49, 6.54)		
Pork (g/week)				
< 250	1.00	1.86 (0.58, 5.93)	0.44 (0.08, 2.53)	0.358

≥ 250	1.67 (1.10, 2.52)	1.36 (0.40, 4.67)		
Fish (times/week)				
≤ 1	1.00	1.36 (0.48, 3.89)	1.34 (0.23, 7.64)	0.746
> 1	0.41 (0.25, 0.67)	0.74 (0.20, 2.82)		
Egg (/week)				
≤ 3	1.00	2.25 (0.63, 8.00)	0.54 (0.10, 2.87)	0.473
> 3	1.72 (1.15, 2.59)	2.10 (0.71, 6.29)		
Fried food (times/month)				
≤ 1	1.00	1.29 (0.48, 3.43)	1.97 (0.32, 12.28)	0.467
> 1	1.50 (0.91, 2.48)	3.80 (0.85, 16.92)		
Sausage (times/month)				
≤ 1	1.00	4.02 (1.22, 13.22)	0.29 (0.03, 3.11)	0.306
> 1	3.87 (1.74, 8.61)	4.51 (0.67, 30.23)		
Chinese pickled sour cabbage (times/month)				
≤ 2	1.00	2.40 (0.80, 9.21)	0.48 (0.10, 2.47)	0.382
> 2	2.02 (1.32, 3.07)	2.33 (0.69, 7.87)		
Leftovers [c] (times/week)				
≤3	1.00	1.36 (0.50, 3.69)	1.09 (0.20, 6.12)	0.920
>3	1.85 (1.21, 2.82)	2.74 (0.69, 10.86)		
Physical exercise				
No	1.00	1.74 (0.52, 5.81)	1.06 (0.01, 86.66)	0.980
Yes	0.05 (0.02, 0.17)	0.10 (0.00, 6.82)		

[a] adjusted for gender, occupation, education, and family history of cancer. [b] $P < 0.05$ in the conditional logistic regression analysis was considered statistically significant. [c] leftovers: leftovers more than 12 hours.

Supplementary Table S6. Interactions Between Five Gene CNVs and Environmental Factors on the Risk of CRC.

**Environmental Factors**	***MDM2***		**Interaction**		***SKP2***		**Interaction**	
	**Wt**	**Del+amp**			**Wt**	**Del+amp**		
	**OR_{eg} (95% CI)[a]**		**OR_i (95% CI)[a]**	***P* Value[b]**	**OR_{eg} (95% CI)[a]**		**OR_i (95% CI)[a]**	***P* Value[b]**
Refined grains (g/day)								
≤ 250	1.00	13.35 (2.13, 89.49)	0.09 (0.04, 2.14)	0.135	1.00	0.36 (0.11, 1.18)	0.26 (0.04, 1.63)	0.150
> 250	2.53 (1.59, 4.02)	**5.44 (1.03, 28.86)**			3.02 (1.80, 5.09)	0.28 (0.07, 1.12)		
Roughage (g/week)								
< 50	1.00	9.37 (0.79, 111.61)	0.67 (0.04, 12.75)	0.791	1.00	0.28 (0.08, 1.00)	0.74 (0.13, 4.30)	0.734
≥ 50	0.62 (0.42, 0.92)	3.90 (0.77, 19.71)			0.62 (0.41, 0.94)	**0.13 (0.04, 0.44)**		
Fruit (times/week)								
< 2	1.00	4.79 (0.71, 32.20)	1.88 (0.11, 32.52)	0.665	1.00	0.09 (0.02, 0.45)	6.10 (0.92, 40.38)	0.061
≥ 2	0.61 (0.40, 0.92)	5.44 (0.66, 45.07)			0.62 (0.40, 0.98)	**0.33 (0.12, 0.96)**		
Fat meat								
No	1.00	11.25 (1.63, 77.25)	0.33 (0.02, 5.09)	0.427	1.00	0.30 (0.10, 0.87)	1.74 (0.24, 8.03)	0.723
Yes	2.30 (1.48, 3.57)	**8.55 (1.22, 59.75)**			2.34 (1.47, 3.74)	0.96 (0.25, 3.79)		
Pork (g/week)								
< 250	1.00	12.40 (1.27, 121.39)	0.23 (0.01, 6.26)	0.380	1.00	0.17 (0.04, 0.68)	2.35 (0.34, 16.07)	0.385
≥ 250	1.65 (1.11, 2.46)	4.63 (0.42, 50.79)			1.51 (1.00, 2.30)	0.61 (0.16, 2.24)		
Fish (times/week)								
≤ 1	1.00	6.36 (0.90, 44.81)	1.53 (0.10, 24.77)	0.765	1.00	0.09 (0.03, 0.31)	**13.62 (1.70, 109.36)**	**0.014**
> 1	0.39 (0.24, 0.64)	3.82 (0.52, 28.34)			0.30 (0.17, 0.52)	0.39 (0.08, 2.00)		
Egg (/week)								
≤ 3	1.00	9.30 (0.66, 131.06)	0.49 (0.02, 10.06)	0.645	1.00	0.36 (0.10, 1.39)	0.80 (0.14, 4.53)	0.799

> 3	1.60 (1.08, 2.37)	**7.33 (1.57, 34.30)**			1.83 (1.21, 2.79)	0.52 (0.18, 1.52)		
Fried food (times/month)								
≤ 1	1.00	4.99 (1.00, 24.85)	1.81 (0.08, 42.46)	0.713	1.00	0.38 (0.16, 0.90)	0.42 (0.03, 5.53)	0.508
> 1	1.44 (0.88, 2.38)	13.02 (0.92, 186.12)			1.44 (0.87, 2.40)	0.23 (0.02, 2.87)		
Sausage (times/month)								
≤ 1	1.00	5.47 (0.95, 31.34)		0.950	1.00	0.14 (0.03, 0.70)		0.552
> 1	3.11 (1.52, 6.37)				3.41 (1.53, 7.57)	2.58 (0.01, 490.04)		
Chinese pickled sour cabbage (times/month)								
≤ 2	1.00	6.41 (1.16, 35.27)	2.10 (0.10, 44.07)	0.633	1.00	0.25 (0.07, 0.83)	1.32 (0.23, 7.51)	0.753
> 2	2.05 (1.35, 3.12)	**27.61 (2.12, 259.81)**			2.01 (1.30, 3.11)	0.67 (0.22, 2.51)		
Leftovers [c] (times/week)								
≤ 3	1.00	3.30 (0.37, 29.54)	4.31 (0.18, 100.81)	0.363	1.00	0.38 (0.14, 0.99)	0.87 (0.14, 5.43)	0.885
> 3	1.87 (1.23, 2.84)	**26.67 (2.62, 271.60)**			2.04 (1.32, 3.15)	0.67 (0.14, 3.23)		
Physical exercise								
No	1.00	3.63 (0.87, 50.68)		0.802	1.00	0.50 (0.13, 1.85)		0.912
Yes	0.04 (0.01, 0.13)				0.06 (0.02, 0.18)			

Continued.

**Environmental Factors**	***FBXW7***		**Interaction**		***NEDD4-1***		**Interaction**	
	**Wt**	**Del+amp**			**Wt**	**Del+amp**		
		**OR_{eg} (95% CI)[a]**	**OR_i (95% CI)[a]**	***P* Value[b]**		**OR_{eg} (95% CI)[a]**	**OR_i (95% CI)[a]**	***P* Value[b]**
Refined grains (g/day)								
≤ 250	1.00	0.87 (0.45, 1.67)	1.43 (0.49, 4.19)	0.519	1.00	1.48 (0.57, 3.86)	0.71 (0.18, 2.81)	0.622
> 250	2.28 (1.37, 3.80)	**2.81 (1.52, 6.86)**			2.63 (1.62, 4.27)	**2.75 (1.00, 7.55)**		
Roughage (g/week)								
< 50	1.00	0.81 (0.38, 1.75)	1.14 (0.41, 3.12)	0.802	1.00	1.21 (0.42, 3.49)	1.10 (0.28, 4.39)	0.891

≥ 50	0.63 (0.41, 0.97)	0.58 (0.29, 1.17)			0.62 (0.41, 0.93)	0.82 (0.34, 2.01)		
Fruit (times/week)								
< 2	1.00	0.87 (0.28, 2.72)	0.98 (0.32, 2.96)	0.969	1.00	0.73 (0.34, 2.55)	1.51 (0.35, 6.47)	0.582
≥ 2		0.40 (0.14, 1.17)			0.60 (0.38, 0.93)	0.83 (0.29, 2.37)		
Fat meat								
No	1.00	0.76 (0.40, 1.47)	1.35 (0.47, 3.82)	0.577	1.00	1.00 (0.38, 2.62)	1.46 (0.37, 5.84)	0.590
Yes	2.23 (1.35, 3.68)	**2.30 (1.03, 5.11)**			2.27 (1.43, 3.60)	**3.33 (1.21, 9.14)**		
Pork (g/week)								
< 250	1.00	0.46 (0.21, 0.99)	**3.13 (1.06, 9.41)**	**0.040**	1.00	1.83 (0.73, 4.61)	0.60 (0.14, 2.62)	0.492
≥ 250	1.34 (0.87, 2.06)	1.92 (0.90, 4.11)			1.66 (1.10, 2.52)	1.82 (0.60, 5.55)		
Fish (times/week)								
≤ 1	1.00	0.76 (0.40, 1.44)	1.54 (0.49, 4.85)	0.463	1.00	1.08 (0.47, 2.49)	3.00 (0.54, 17.72)	0.414
> 1	0.37 (0.22, 0.63)	0.43 (0.17, 1.08)			0.39 (0.24, 0.65)	0.82 (0.24, 2.82)		
Egg (/week)								
≤ 3	1.00	0.94 (0.41, 2.14)	0.85 (0.30, 2.44)	0.758	1.00	0.94 (0.41, 2.14)	0.28 (0.06, 1.19)	0.084
> 3	1.71 (1.10, 2.64)	1.36 (0.67, 2.74)			1.70 (1.10, 2.64)	1.37 (0.47, 2.74)		
Fried food (times/month)								
≤ 1	1.00	0.71 (0.40, 1.28)	4.65 (0.50, 5.42)	0.413	1.00	0.71 (0.38, 1.28)	1.21 (0.23, 6.34)	0.824
> 1	1.32 (0.78, 2.23)	1.54 (0.56, 4.29)			1.32 (0.78, 2.23)	1.54 (0.56, 4.29)		
Sausage (times/month)								
≤ 1	1.00	1.58 (0.70, 3.60)	0.43 (0.07, 2.58)	0.356	1.00	1.58 (0.70, 3.60)		0.883
> 1	3.84 (1.67, 8.86)	2.62 (0.57, 11.98)			3.84 (1.66, 8.87)	2.62 (0.57, 11.98)		
Chinese pickled sour cabbage (times/month)								
≤ 2	1.00	0.80 (0.38, 1.66)	1.79 (0.43, 3.29)	0.738	1.00	0.80 (0.38, 1.66)	1.63 (0.40, 6.59)	0.496
> 2	1.96 (1.27, 3.04)	1.86 (0.88, 3.91)			1.96 (1.27, 3.04)	1.86 (0.89, 3.91)		
Leftovers [c] (times/week)								

≤ 3	1.00	0.70 (0.34, 1.43)	1.38 (0.50, 3.80)	0.530	1.00	0.70 (0.34, 1.43)	0.98 (0.24, 4.05)	0.972
> 3	1.83 (1.16, 2.88)	1.77 (0.85, 3.70)			1.83 (0.16, 2.88)	1.77 (0.85, 3.70)		
Physical exercise								
No	1.00	1.58 (0.66, 3.78)	0.65 (0.10, 4.47)	0.662	1.00	1.58 (0.66, 3.78)	10.27 (0.60, 177.48)	0.109
Yes	0.06 (0.02, 0.20)	**0.06 (0.01, 0.31)**			0.06 (0.02, 0.20)	**0.06 (0.01, 0.31)**		

Continued.

**Environmental Factors**	***β-TRCP***		**Interaction**	
	**Wt**	**Del+amp**		
	**OR_{eg} (95% CI)[a]**		**OR_i (95% CI)[a]**	***P* Value[b]**
Refined grains (g/day)				
≤ 250	1.00	1.76 (0.65, 4.78)	0.72 (0.14, 3.60)	0.684
> 250	2.60 (1.60, 4.23)	3.27 (0.93, 11.52)		
Roughage (g/week)				
< 50	1.00	0.58 (0.18, 1.86)	**4.74 (0.99, 22.61)**	**0.051**
≥ 50	0.55 (0.37, 0.82)	1.51 (0.54, 4.18)		
Fruit (times/week)				
< 2	1.00	2.10 (0.64, 6.80)	0.53 (0.11, 2.53)	0.424
≥ 2	0.63 (0.41, 0.98)	0.70 (0.26, 1.85)		
Fat meat				
No	1.00	2.49 (0.86, 7.19)	0.26 (0.05, 1.27)	0.096
Yes	2.34 (1.51, 3.63)	1.50 (0.46, 4.86)		
Pork (g/week)				
< 250	1.00	1.72 (0.59, 4.99)	0.43 (0.09, 2.10)	0.296
≥ 250	1.68 (1.11, 5.53)	1.23 (0.40, 3.77)		
Fish (times/week)				

≤ 1	1.00	1.30 (0.47, 3.59)	1.28 (0.26, 6.27)	0.763
> 1	0.40 (0.24, 0.67)	0.67 (0.21, 2.07)		
Egg (/week)				
≤ 3	1.00	2.91 (0.65, 8.07)	0.43 (0.09, 2.08)	0.292
> 3	1.74 (1.16, 2.62)	1.70 (0.66, 4.40)		
Fried food (times/month)				
≤ 1	1.00	1.12 (0.47, 2.68)	2.28 (0.39, 13.36)	0.361
> 1	1.48 (0.90, 2.46)	3.80 (0.86, 16.85)		
Sausage (times/month)				
≤ 1	1.00	3.00 (1.06, 8.55)	1.30 (0.75, 2.23)	0.352
> 1	3.67 (1.67, 8.03)	4.38 (0.66, 29.11)		
Chinese pickled sour cabbage (times/month)				
≤ 2	1.00	1.82 (0.65, 5.10)	0.62 (0.14, 2.83)	0.541
> 2	1.97 (1.29, 3.00)	2.25 (0.75, 6.71)		
Leftovers [c] (times/week)				
≤3	1.00	1.11 (0.43, 2.84)	1.46 (0.30, 7.19)	0.639
>3	1.82 (1.19, 2.79)	**2.60 (0.86, 0.25)**		
Physical exercise				
No	1.00	1.46 (0.51, 4.22)	1.26 (0.02, 98.52)	0.918
Yes	0.05 (0.02, 0.17)	0.10 (0.00, 6.74)		

[a] adjusted for gender, occupation, education, and family history of cancer. [b] $P < 0.05$ in the conditional logistic regression analysis was considered statistically significant. [c] leftovers: leftovers more than 12 hours

Supplementary Table S7. Interactions between five gene amplifications and environmental factors on the risk of CRC after interpolation.

Environmental Factors	*MDM2*		Interaction		*SKP2*		Interaction	
	**Del+Wt**	**Amp**			**Del+Wt**	**Amp**		
	**OR$_{eg}$ (95% CI)[a]**		**OR$_{i}$ (95% CI)[a]**	***P* Value[b]**	**OR$_{eg}$ (95% CI)[a]**		**OR$_{i}$ (95% CI)[a]**	***P* Value[b]**
Refined grains (g/day)								
≤ 250	1.00	12.54 (1.65, 95.54)		0.934	1.00	0.49 (0.14, 1.68)	0.78 (0.09, 6.95)	0.821
> 250	2.48 (1.74, 3.52)				2.59 (1.78, 3.78)	0.98 (0.17, 5.67)		
Roughage (g/week)								
< 50	1.00			0.757	1.00	0.62 (0.17, 2.26)	0.51 (0.07, 3.44)	0.485
≥ 50	0.80 (0.58, 1.09)	4.38 (0.50, 38.52)			0.75 (0.54, 1.04)	**0.24 (0.06, 0.98)**		
Fruit (times/week)								
< 2	1.00			0.812	1.00	0.21 (0.05, 0.97)	4.11 (0.52, 32.68)	0.181
≥ 2	0.83 (0.62, 1.12)	8.88 (1.01, 78.16)			0.81 (0.59, 1.10)	0.69 (0.19, 2.57)		
Fat meat								
No	1.00	11.34 (1.10, 116.89)		0.834	1.00	0.62 (0.18, 2.18)	0.41 (0.04, 4.41)	0.456
Yes	1.89 (1.35, 2.64)				2.07 (1.46, 2.92)	0.53 (0.08, 3.36)		
Pork (g/week)								
< 250	1.00	8.90 (0.85, 93.27)	2.53 (0.05, 119.35)	0.637	1.00	0.34 (0.09, 1.38)	1.72 (0.22, 13.48)	0.607
≥ 250	1.57 (1.15, 2.15)	**35.57 (1.69, 740.79)**			1.49 (1.03, 1.98)	0.88 (0.20, 3.79)		
Fish (times/week)								
≤ 1	1.00			0.800	1.00	0.37 (0.11, 1.26)	3.58 (0.30, 43.23)	0.312
> 1	0.29 (0.19, 0.43)	3.57 (0.35, 36.42)			0.25 (0.16, 0.39)	0.34 (0.05, 2.39)		
Egg (/week)								
≤ 3	1.00	10.40 (0.73, 149.20)	1.16 (0.03, 53.95)	0.939	1.00	0.45 (0.09, 2.18)	0.93 (0.12, 7.37)	0.945
> 3	1.51 (1.11, 2.06)	**18.25 (1.08, 309.28)**			1.56 (1.13, 2.17)	0.66 (0.18, 2.36)		

Fried food (times/month)								
≤ 1	1.00	8.52 (0.77, 94.71)	1.82 (0.04, 82.88)	0.758	1.00	0.44 (0.16, 1.26)	1.38 (0.09, 19.74)	0.823
> 1	1.71 (1.17, 2.51)	**26.60 (1.08, 309.28)**			1.65 (1.12, 2.44)	0.99 (0.09, 11.59)		
Sausage (times/month)								
≤ 1	1.00	12.28 (1.66, 90.79)		0.897	1.00	0.43 (0.13, 1.47)	0.88 (0.03, 30.50)	0.940
> 1	1.98 (1.28, 3.06)				2.12 (1.36, 3.31)	0.81 (0.39, 16.67)		
Chinese pickled sour cabbage (times/month)								
≤ 2	1.00	14.00 (1.39, 141.20)	2.03 (0.04, 91.01)	0.715	1.00	0.30 (0.07, 1.24)	2.04 (0.27, 15.25)	0.486
> 2	2.30 (1.66, 3.18)	**65.29 (3.04, 1403.85)**			2.22 (1.59, 3.09)	1.38 (0.34, 5.59)		
Leftovers [c] (times/week)								
≤ 3	1.00	2.11 (0.12, 38.71)		0.685	1.00	0.35 (0.10, 1.25)	2.46 (0.25, 24.32)	0.438
> 3	1.46 (1.04, 2.06)				1.47 (1.03, 2.08)	1.26 (0.21, 7.59)		
Physical exercise								
No	1.00	27.68 (1.79, 429.02)	0.28 (0.01, 14.67)	0.524	1.00	0.36 (0.12, 1.10)		0.993
Yes	0.24 (0.16, 0.38)	1.85 (0.11, 31.37)			0.23 (0.13, 0.42)			

Continued

**Environmental Factors**	***FBXW7***		**Interaction**		***NEDD4-1***		**Interaction**	
	**Del+wt**	**Amp**			**Del+wt**	**Amp**		
	**OR_{eg} (95% CI)[a]**		**OR_i (95% CI)[a]**	***P* Value[b]**	**OR_{eg} (95% CI)[a]**		**OR_i (95% CI)[a]**	***P* Value[b]**
Refined grains (g/day)								
≤ 250	1.00	0.56 (0.30, 1.14)	1.25 (0.39, 4.04)	0.709	1.00	2.33 (1.03, 5.27)	0.40 (0.12, 1.32)	0.131
> 250	2.51 (1.69, 3.73)	1.76 (0.74, 4.20)			2.80 (1.93, 4.08)	**2.58 (1.07, 6.27)**		
Roughage (g/week)								
< 50	1.00	0.60 (0.30, 1.24)	2.29 (0.74, 7.05)	0.151	1.00	1.30 (0.62, 2.71)	1.45 (0.43, 4.91)	0.551
≥ 50	0.57 (0.41, 0.80)	0.79 (0.33, 1.86)			0.61 (0.44, 0.84)	1.14 (0.42, 3.05)		

Fruit (times/week)								
< 2	1.00	0.20 (0.23, 1.09)	**1.63 (1.07, 2.48)**	**0.023**	1.00	1.30 (0.58, 2.93)	1.45 (0.85, 2.21)	0.084
≥ 2	0.81 (0.59, 1.10)	0.75 (0.36, 1.55)			0.81 (0.59, 1.12)	1.60 (0.70, 3.65)		
Fat meat								
No	1.00	0.59 (0.28, 1.24)	1.51 (0.50, 4.53)	0.464	1.00	1.91 (0.85, 4.28)	0.68 (0.22, 2.11)	0.499
Yes	1.96 (1.37, 2.78)	1.74 (0.77, 3.94)			1.93 (1.37, 2.93)	**2.48 (1.04, 5.90)**		
Pork (g/week)								
< 250	1.00	0.62 (0.30, 1.30)	1.26 (0.45, 3.50)	0.662	1.00	2.09 (0.94, 4.64)	0.59 (0.17, 1.97)	0.386
≥ 250	1.52 (1.09, 2.11)	1.19 (0.56, 2.53)			1.58 (1.14, 2.20)	1.94 (0.78, 4.81)		
Fish (times/week)								
≤ 1	1.00	0.57 (0.29, 1.12)	1.51 (0.40, 5.67)	0.544	1.00	1.24 (0.62, 2.48)	2.11 (0.55, 8.03)	0.275
> 1	0.26 (0.17, 0.40)	**0.22 (0.07, 0.68)**			0.29 (0.19, 0.43)	0.74 (0.23, 2.37)		
Egg (/week)								
≤ 3	1.00	0.73 (0.32, 1.65)	1.00 (0.34, 2.97)	0.996	1.00	4.15 (1.66, 10.36)	**0.21 (0.06, 0.68)**	**0.010**
> 3	1.44 (1.03, 2.02)	1.05 (0.50, 2.24)			1.75 (1.25, 2.44)	1.49 (0.68, 3.29)		
Fried food (times/month)								
≤ 1	1.00	0.53 (0.29, 0.99)	2.92 (0.81, 10.46)	0.100	1.00	1.98 (1.03, 3.84)	0.51 (0.13, 1.99)	0.334
> 1	1.54 (1.03, 2.36)	2.41 (0.81, 7.19)			1.85 (1.24, 2.77)	1.87 (0.58, 6.08)		
Sausage (times/month)								
≤ 1	1.00	0.77 (0.42, 1.39)	0.68 (0.18, 2.57)	0.570	1.00	1.76 (0.92, 3.34)	0.85 (0.16, 4.63)	0.848
> 1	2.09 (1.30, 3.35)	1.09 (0.34, 3.53)			1.89 (1.19, 2.98)	2.82 (0.66, 12.00)		
Chinese pickled sour cabbage (times/month)								
≤ 2	1.00	0.88 (0.43, 1.78)	0.63 (0.22, 1.84)	0.391	1.00	0.93 (0.42, 2.06)	2.84 (0.81, 9.92)	0.101
> 2	2.35 (1.67, 3.29)	1.30 (0.57, 2.97)			1.99 (1.42, 2.78)	**5.26 (2.07, 13.32)**		
Leftovers [c] (times/week)								
≤ 3	1.00	0.63 (0.31, 1.27)	1.25 (0.41, 3.79)	0.694	1.00	1.56 (0.77, 3.13)	1.39 (0.38, 5.06)	0.617

> 3	1.47 (1.02, 2.11)	1.15 (0.50, 2.68)			1.47 (1.04, 2.08)	3.19 (0.84, 3.75)		
Physical exercise								
No	1.00	0.85 (0.41, 1.75)	0.58 (0.04, 9.40)	0.677	1.00	1.77 (0.84, 3.75)	1.30 (0.25, 6.64)	0.748
Yes	0.25 (0.14, 0.42)	0.12 (0.01, 1.30)			0.22 (0.13, 0.36)	0.50 (0.12, 2.14)		

Continued.

Environmental Factors	***β-TRCP***		**Interaction**	
	**Del+wt**	**Amp**		
	**OR_{eg} (95% CI)[a]**		**OR_i (95% CI)[a]**	***P* Value[b]**
Refined grains (g/day)				
≤ 250	1.00	2.51 (0.98, 6.43)	0.42 (0.10, 1.76)	0.233
> 250	2.76 (1.90, 4.03)	2.87 (0.99, 8.27)		
Roughage (g/week)				
< 50	1.00	1.94 (0.73, 5.15)	0.80 (0.20, 3.23)	0.757
≥ 50	0.57 (0.41, 0.79)	0.88 (0.33, 2.32)		
Fruit (times/week)				
< 2	1.00	1.92 (0.67, 5.46)	0.77 (0.55, 1.57)	0.691
≥ 2	0.93 (0.61, 1.14)	1.32 (0.54, 3.23)		
Fat meat				
No	1.00	2.45 (0.95, 6.32)	0.48 (0.12, 1.90)	0.295
Yes	2.08 (1.48, 2.92)	2.44 (0.88, 6.74)		
Pork (g/week)				
< 250	1.00	2.44 (0.88, 6.81)	0.52 (0.13, 2.13)	0.363
≥ 250	1.57 (1.13, 2.17)	1.99 (0.97, 5.15)		
Fish (times/week)				
≤ 1	1.00	3.05 (0.77, 12.01)	1.74 (0.41, 7.38)	0.452

> 1	0.41 (0.26, 0.65)	1.03 (0.46, 2.31)		
Egg (/week)				
≤ 3	1.00	1.48 (0.45, 5.38)	0.81 (0.20, 3.31)	0.770
> 3	1.66 (1.18, 2.35)	**3.12 (1.32, 7.38)**		
Fried food (times/month)				
≤ 1	1.00	1.62 (0.74, 3.57)	1.45 (0.30, 6.94)	0.645
> 1	1.75 (1.17, 2.62)	**4.10 (1.06, 15.86)**		
Sausage (times/month)				
≤ 1	1.00	2.53 (0.53, 12.15)	0.66 (0.10, 4.60)	0.670
> 1	1.10 (1.28, 3.11)	2.06 (0.86, 4.96)		
Chinese pickled sour cabbage (times/month)				
≤ 2	1.00	2.06 (0.86, 4.95)	0.83 (0.21, 3.27)	0.785
> 2	2.18 (1.54, 3.12)	**3.70 (1.23, 11.17)**		
Leftovers [c] (times/week)				
≤ 3	1.00	1.64 (0.71, 3.78)	0.93 (0.22, 3.85)	0.916
> 3	1.48 (1.04, 2.10)	2.24 (0.72, 6.95)		
Physical exercise				
No	1.00	1.74 (0.75, 4.04)	0.51 (0.02, 10.69)	0.644
Yes	0.26 (0.16, 0.42)	0.23 (0.02, 3.46)		

CI, confidence interval; CRC, colorectal cancer; OR, odds ratio. [a] adjusted for gender, occupation, education, and family history of cancer. [b] $P < 0.05$ in the conditional logistic regression analysis was considered statistically significant. [c] leftovers: leftovers more than 12 hours

Supplementary Table S8. Interactions between five gene CNVs and environmental factors on the risk of CRC after interpolation.

Environmental Factors	*MDM2*		Interaction		*SKP2*		Interaction	
	Wt	Del+amp			Wt	Del+amp		
	OR_{eg} (95% CI)[a]		OR_i (95% CI)[a]	*P* Value[b]	OR_{eg} (95% CI)[a]		OR_i (95% CI)[a]	*P* Value[b]
Refined grains (g/day)								
≤ 250	1.00	1.02 (0.79, 1. 30)	**0.07 (0.01, 0.84)**	**0.036**	1.00	0.55 (0.24, 1.23)	0.51 (0.15, 1.79)	0.295
> 250	0.07 (0.02, 60.21)	0.85 (0.71, 1.02)			2.87 (1.93, 4.28)	0.80 (0.32, 2.00)		
Roughage (g/week)								
< 50	1.00	5.14 (0.98, 26.73)	0.82 (0.09, 7.26)	0.861	1.00	0.35 (0.15, 0.85)	2.13 (0.59, 7.63)	0.247
≥ 50	0.55 (0.40, 0.76)	2.33 (0.52, 10.51)			0.55 (0.39, 0.77)	0.41 (0.16, 1.04)		
Fruit (times/week)								
< 2	1.00	2.72 (0.58, 12.84)	2.07 (0.87, 4.93)	0.101	1.00	0.41 (0.46, 1.03)	1.28 (0.77, 2.13)	0.343
≥ 2	0.81 (0.60, 1.10)	5.89 (1.06, 32.72)			0.83 (0.60, 1.14)	0.48 (0.21, 1.06)		
Fat meat								
No	1.00	6.67 (1.14, 39.11)	0.38 (0.04, 3.93)	0.420	1.00	0.45 (0.20, 1.00)	1.18 (0.31, 4.47)	0.812
Yes	1.90 (1.36, 2.67)	**4.87 (1.06, 22.32)**			2.02 (1.41, 2.85)	1.07 (0.38, 2.96)		
Pork (g/week)								
< 250	1.00	3.21 (0.91, 11.25)	2.28 (0.20, 26.37)	0.511	1.00	0.25 (0.10, 0.65)	3.59 (0.90, 14.29)	0.069
≥ 250	1.52 (1.12, 2.08)	**11.10 (1.32, 93.17)**			1.43 (1.03, 1.98)	1.30 (0.53, 3.22)		
Fish (times/week)								
≤ 1	1.00	6.43 (1.28, 32.42)	0.60 (0.06, 5.79)	0.657	1.00	0.29 (0.13, 0.65)	3.62 (0.76, 17.14)	0.104
> 1	0.29 (0.20, 0.44)	1.12 (0.23, 5.43)			0.22 (0.14, 0.36)	**0.23 (0.07, 0.75)**		
Egg (/week)								
≤ 3	1.00	4.45 (0.53, 37.24)	0.78 (0.07, 9.47)	0.847	1.00	0.35 (0.13, 0.95)	1.67 (0.45, 6.17)	0.443
> 3	1.50 (1.09, 2.05)	**5.21 (1.37, 19.76)**			1.52 (1.09, 2.13)	0.88 (0.39, 1.96)		

Fried food (times/month)								
≤ 1	1.00	2.92 (0.86, 9.88)	6.33 (0.31, 130.83)	0.232	1.00	0.55 (0.29, 1.05)	0.57 (0.12, 3.05)	0.507
> 1	1.68 (1.14, 2.47)	**30.98 (1.95, 492.22)**			1.68 (1.13, 2.49)	0.52 (0.11, 2.51)		
Sausage (times/month)								
≤ 1	1.00	4.467 (1.34, 14.53)		0.915	1.00	0.39 (0.18, 0.84)	2.53 (0.45, 14.29)	0.289
> 1	2.00 (1.30, 3.07)				1.97 (1.24, 3.14)	1.93 (0.46, 8.10)		
Chinese pickled sour cabbage (times/month)								
≤ 2	1.00	3.35 (0.89, 12.64)	8.04 (0.51, 127.66)	0.140	1.00	0.63 (0.27, 1.47)	0.63 (0.18, 2.19)	0.471
> 2	2.23 (1.61, 3.08)	**59.90 (4.91, 729.84)**			2.27 (1.62, 3.18)	0.91 (0.37, 2.23)		
Leftovers [c] (times/week)								
≤ 3	1.00	2.45 (0.41, 14.64)	2.68 (0.24, 30.20)	0.425	1.00	0.49 (0.23, 1.06)	1.06 (0.29, 3.80)	0.934
> 3	1.47 (1.40, 2.07)	**9.64 (1.78, 52.11)**			1.48 (1.04, 2.11)	0.77 (0.28, 2.14)		
Physical exercise								
No	1.00	4.81 (1.18, 19.55)	0.87 (0.06, 11.92)	0.916	1.00	0.39 (0.18, 0.84)	1.61 (0.18, 13.98)	0.659
Yes	0.24 (0.16, 0.39)	1.02 (0.12, 8.40)			0.23 (0.13, 0.39)	0.14 (0.02, 1.42)		

Continued

**Environmental Factors**	***FBXW7***		**Interaction**		***β-TRCP***		**Interaction**	
	**Wt**	**Del+amp**			**Wt**	**Del+amp**		
	**OR_{eg} (95% CI)[a]**		**OR_i (95% CI)[a]**	***P* Value[b]**	**OR_{eg} (95% CI)[a]**		**OR_i (95% CI)[a]**	***P* Value[b]**
Refined grains (g/day)								
≤ 250	1.00	1.10 (0.66, 1.83)	0.75 (0.32, 1.78)	0.513	1.00	1.93 (0.84, 4.44)	0.47 (0.12, 1.78)	0.263
> 250	2.65 (1.76, 3.97)	**2.17 (1.11, 4.27)**			2.80 (1.91, 4.10)	2.52 (0.93, 6.87)		
Roughage (g/week)								
< 50	1.00	1.16 (0.63, 2.13)	0.72 (0.32, 1.63)	0.428	1.00	0.66 (0.25, 1.77)	3.10 (0.87, 11.07)	0.082
≥ 50	0.80 (0.56, 1.15)	0.67 (0.38, 1.17)			0.69 (0.49, 0.97)	1.42 (0.62, 3.24)		

Fruit (times/week)								
< 2	1.00	0.84 (0.45, 1.57)	1.29 (0.56, 3.00)	0.552	1.00	1.56 (0.62, 3.90)	0.78 (0.23, 2.67)	0.688
≥ 2	0.82 (0.59, 1.14)	0.89 (0.51, 1.54)			0.84 (0.61, 1.14)	1.01 (0.45, 2.27)		
Fat meat								
No	1.00	0.99 (0.58, 1.68)	1.01 (0.45, 2.26)	0.984	1.00	2.22 (0.93, 5.33)	0.36 (0.10, 1.25)	0.106
Yes	2.04 (1.41, 3.00)	**2.03 (1.09, 3.81)**			2.14 (1.52, 3.01)	1.69 (0.69, 4.17)		
Pork (g/week)								
< 250	1.00	0.77 (0.44, 1.35)	1.58 (0.69, 3.64)	0.279	1.00	1.83 (0.73, 4.57)	0.52 (0.15, 1.85)	0.316
≥ 250	1.44 (1.02, 2.03)	1.75 (0.96, 3.19)			1.57 (1.13, 2.16)	1.50 (0.64, 3.51)		
Fish (times/week)								
≤ 1	1.00	0.82 (0.49, 1.37)	1.80 (0.68, 4.73)	0.236	1.00	1.23 (0.53, 2.85)	1.41 (0.37, 5.31)	0.611
> 1	0.25 (0.16, 0.38)	**0.36 (0.17, 0.77)**			0.28 (0.19, 0.43)	0.49 (0.19, 1.27)		
Egg (/week)								
≤ 3	1.00	1.05 (0.54, 2.03)	0.91 (0.38, 2.16)	0.832	1.00	1.53 (0.58, 4.04)	0.75 (0.21, 2.69)	0.657
> 3	1.49 (1.04, 2.12)	1.42 (0.81, 2.49)			1.47 (1.06, 2.04)	1.69 (0.76, 3.79)		
Fried food (times/month)								
≤ 1	1.00	0.91 (0.58, 1.44)	1.36 (0.52, 3.57)	0.531	1.00	1.21 (0.60, 2.44)	1.68 (0.39, 7.23)	0.489
> 1	1.65 (1.08, 2.53)	2.06 (0.90, 4.70)			1.72 (1.15, 2.58)	3.48 (0.97, 12.49)		
Sausage (times/month)								
≤ 1	1.00	0.96 (0.61, 1.50)	1.06 (0.37, 3.08)	0.913	1.00	1.40 (0.67, 2.91)	0.89 (0.13, 6.04)	0.902
> 1	2.00 (1.19, 3.35)	2.03 (0.84, 4.92)			1.96 (1.21, 3.04)	2.43 (0.50, 11.81)		
Chinese pickled sour cabbage (times/month)								
≤ 2	1.00	0.85 (0.48, 1.53)	1.28 (0.56, 2.92)	0.563	1.00	1.53 (0.67, 3.47)	0.82 (0.24, 2.83)	0.748
> 2	2.18 (1.54, 3.10)	**2.38 (1.31, 4.33)**			2.16 (1.55, 2.99)	**2.69 (1.02, 7.10)**		
Leftovers [c] (times/week)								
≤ 3	1.00	0.79 (0.46, 1.35)	1.64 (0.72, 3.74)	0.235	1.00	1.21 (0.58, 2.61)	1.04 (0.29, 3.81)	0.948

> 3	1.37 (0.95, 1.98)	1.78 (0.94, 3.53)			1.48 (1.04, 2.10)	1.86 (0.68, 5.09)		
Physical exercise								
No	1.00	1.09 (0.64, 1.85)	0.78 (0.19, 3.18)	0.707	1.00	1.35 (0.64, 2.85)	0.48 (0.03, 8.08)	0.586
Yes	0.25 (0.14, 0.42)	**0.21 (0.06, 0.72)**			0.26 (0.17, 0.42)	0.17 (0.01, 2.14)		

Continued

**Environmental Factors**	***NEDD4-1***		**Interaction**	
	**Wt**	**Del+amp**		
	**OR_{eg} (95% CI)[a]**		**OR_i (95% CI)[a]**	***P* Value[b]**
Refined grains (g/day)				
≤ 250	1.00	2.06 (0.98, 4.36)	0.38 (0.12, 1.18)	0.095
> 250	2.85 (1.96, 4.16)	2.24 (0.96, 5.22)		
Roughage (g/week)				
< 50	1.00	2.00 (0.83, 4.81)	0.60 (0.20, 1.83)	0.372
≥ 50	0.79 (0.57, 1.10)	0.96 (0.48, 1.92)		
Fruit (times/week)				
< 2	1.00	1.08 (0.51, 2.32)	1.68 (0.58, 4.85)	0.340
≥ 2	0.80 (0.58, 1.10)	1.45 (0.67, 3.17)		
Fat meat				
No	1.00	1.59 (0.76, 3.31)	0.76 (0.25, 2.28)	0.626
Yes	1.93 (1.36, 2.74)	**2.33 (1.00, 5.44)**		
Pork (g/week)				
< 250	1.00	1.65 (0.80, 3.40)	0.74 (0.24, 2.30)	0.599
≥ 250	1.56 (1.12, 2.15)	1.89 (0.79, 4.53)		
Fish (times/week)				
≤ 1	1.00	1.25 (0.64, 2.47)	1.45 (0.43, 4.93)	0.548

> 1	0.29 (0.19, 0.43)	0.53 (0.19, 1.44)		
Egg (/week)				
≤ 3	1.00	3.06 (1.32, 7.07)	**0.27 (0.09, 0.84)**	**0.023**
> 3	1.73 (1.24, 2.42)	1.44 (0.68, 3.04)		
Fried food (times/month)				
≤ 1	1.00	1.68 (0.91, 3.10)	0.60 (0.16, 2.28)	0.451
> 1	1.83 (1.23, 2.74)	1.84 (0.57, 5.93)		
Sausage (times/month)				
≤ 1	1.00	1.50 (0.83, 2.71)	1.00 (0.19, 5.35)	0.996
> 1	1.86 (1.18, 2.95)	2.80 (0.66, 11.99)		
Chinese pickled sour cabbage (times/month)				
≤ 2	1.00	0.81 (0.38, 1.75)	2.56 (0.79, 8.22)	0.115
> 2	1.99 (1.42, 2.78)	**4.14 (1.79, 9.58)**		
Leftovers [c] (times/week)				
≤ 3	1.00	1.28 (0.66, 2.47)	1.71 (0.51, 5.72)	0.384
> 3	1.45 (1.03, 2.05)	**3.17 (1.13, 8.93)**		
Physical exercise				
No	1.00	1.47 (0.76, 2.88)	1.49 (0.31, 7.20)	0.611
Yes	0.21 (0.13, 0.35)	0.47 (0.11, 2.02)		

CI, confidence interval; CRC, colorectal cancer; OR, odds ratio. [a] adjusted for gender, occupation, education, and family history of cancer. [b] $P < 0.05$ in the conditional logistic regression analysis was considered statistically significant. [c] leftovers: leftovers more than 12 hours

Supplementary Figure S1. Copy number produced by CopyCaller v2.0 for selected cases and controls for different genes.

The red arrow respect the Samples used as calibrators.

FBXW7:

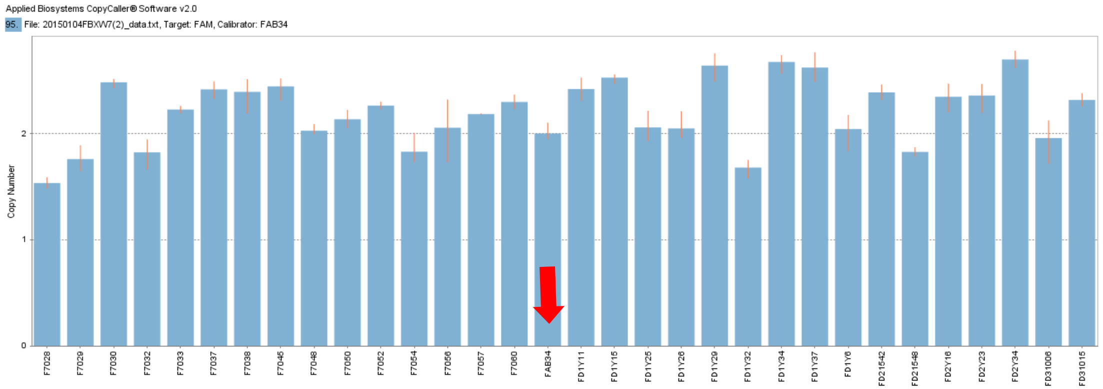

MDM2:

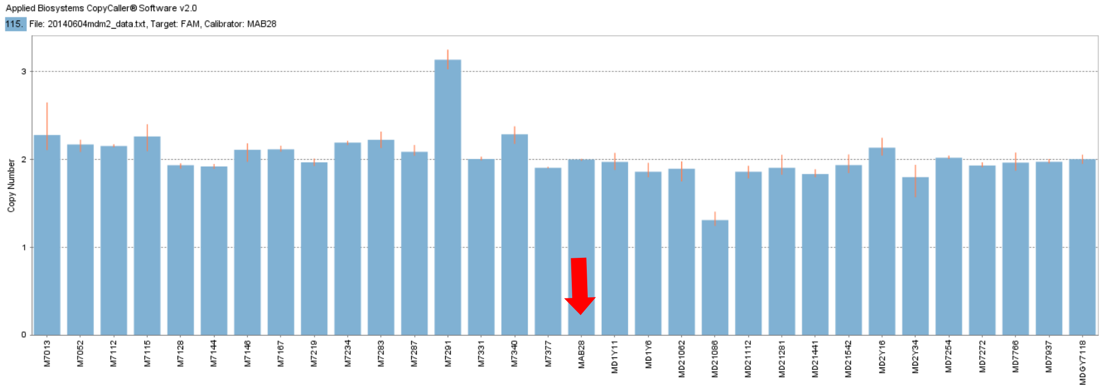

SKP2:

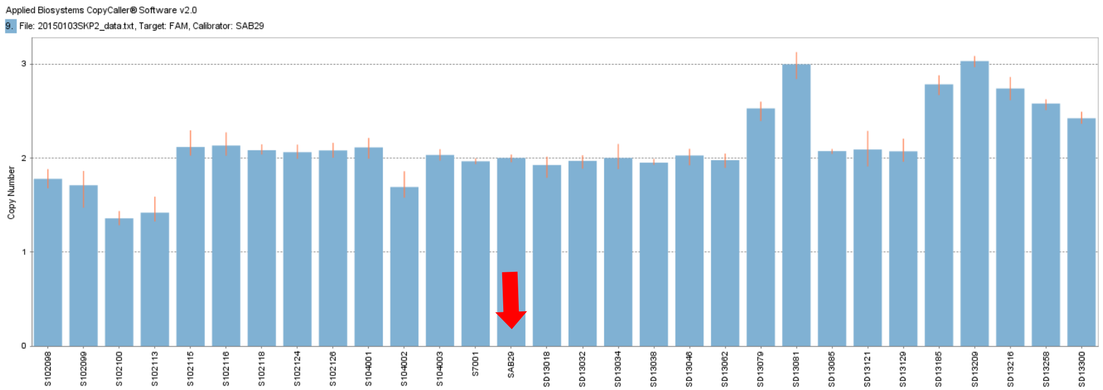

β-TRCP:

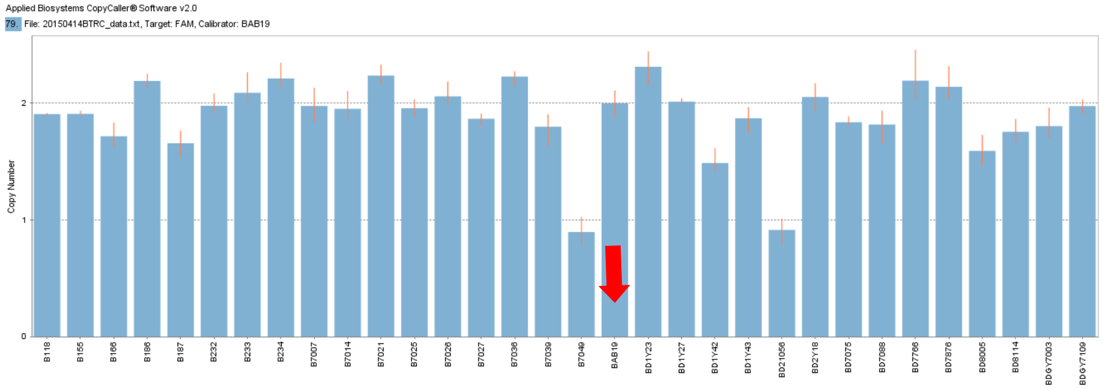

NEDD4-1:

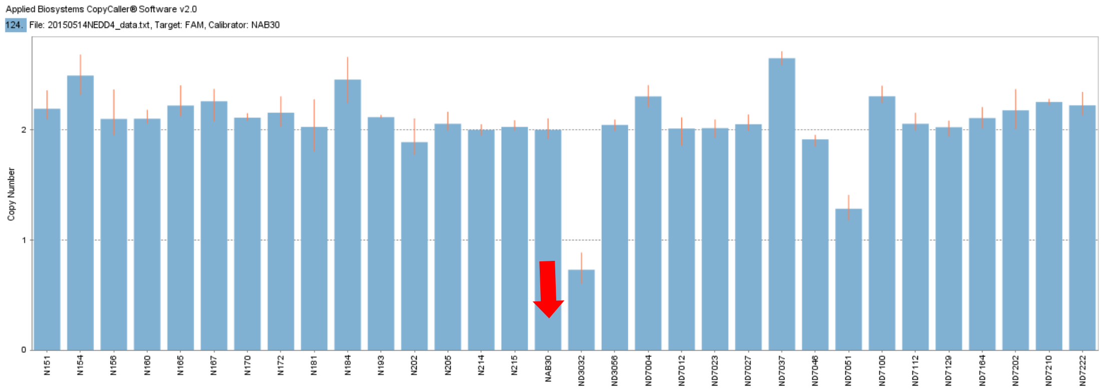

Supplementary Figure S2 Kaplan–Meier curves of overall survival (OS) according to the five genes CNVs in patients with CRC. a *FBXW7* CNVs in CRC; b *MDM2* CNVs in CRC; c *SKP2* CNVs in CRC; d *β-TRCP* CNVs in CRC; e *NEDD4-1* CNVs in CRC.

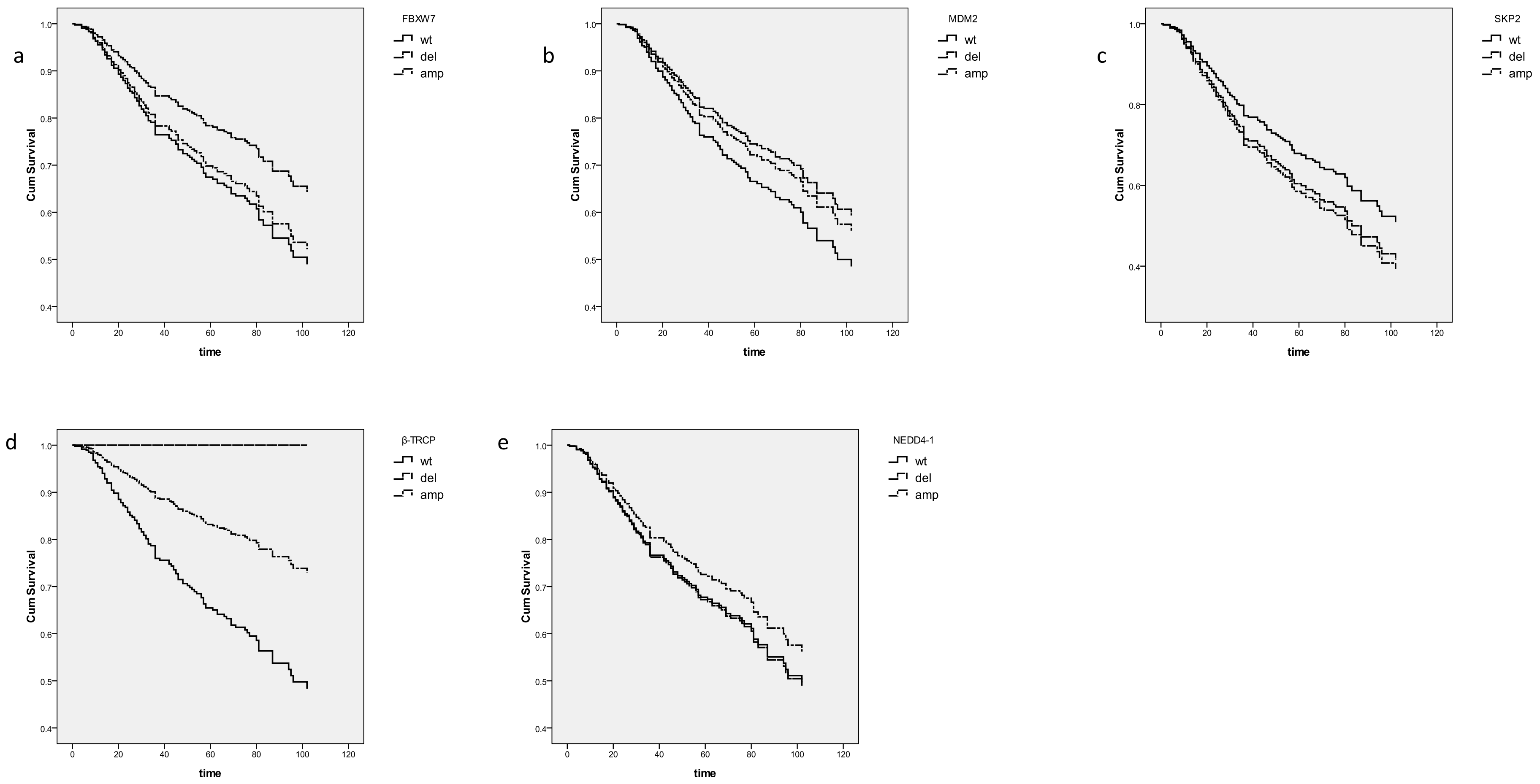

Supplementary Figure S3 Kaplan–Meier curves of overall survival (OS) according to the five genes amplifications in the combined model in patients with CRC. a *FBXW7* CNVs in CRC; b *MDM2* CNVs in CRC; c *SKP2* CNVs in CRC; d *β-TRCP* CNVs in CRC; e *NEDD4-1* CNVs in CRC.

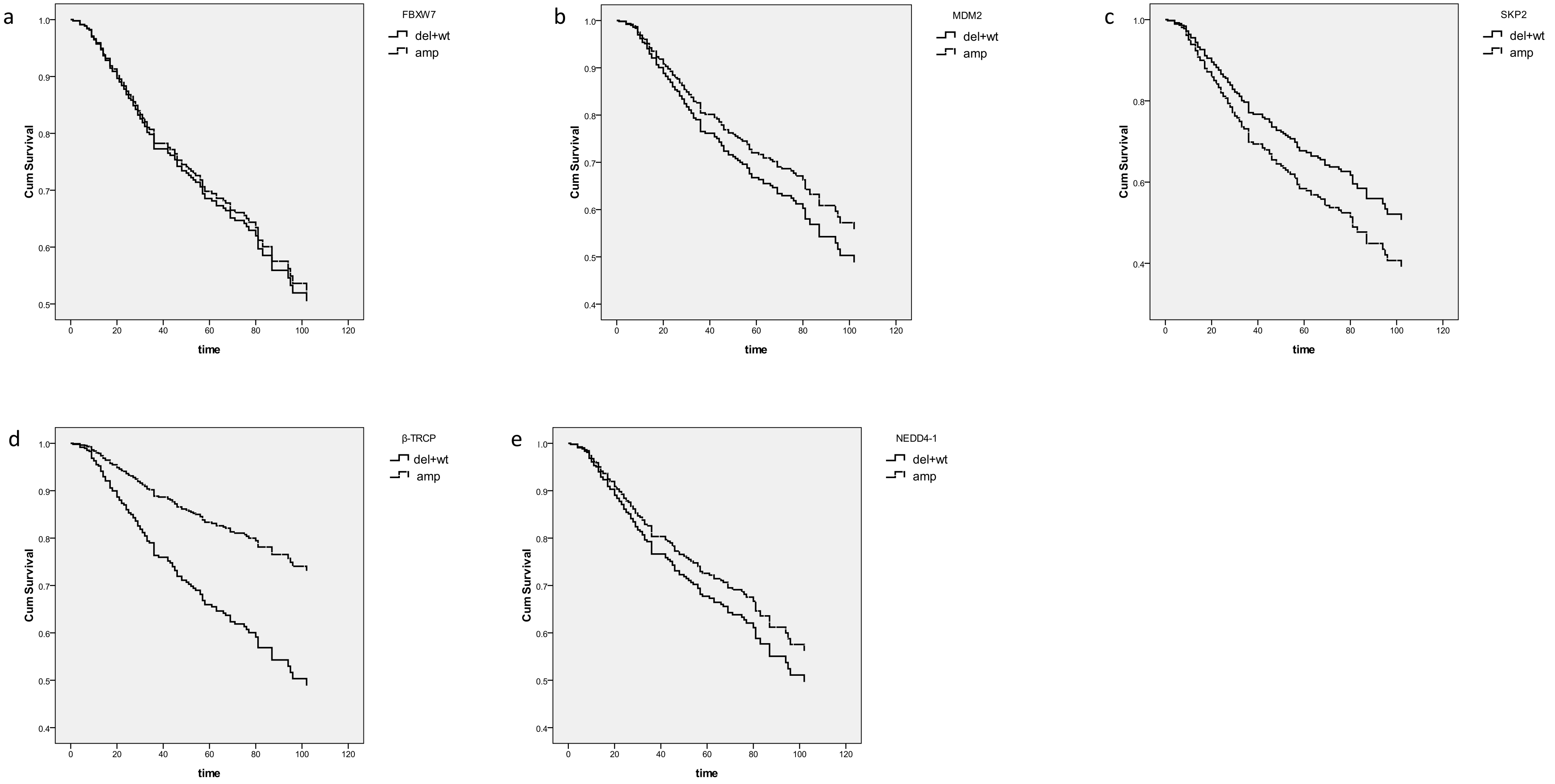

Supplementary Figure S4 Kaplan–Meier curves of overall survival (OS) according to the five genes CNVs in combined model in patients with CRC. a *FBXW7* CNVs in CRC; b *MDM2* CNVs in CRC; c *SKP2* CNVs in CRC; d *β-TRCP* CNVs in CRC; e *NEDD4-1* CNVs in CRC.

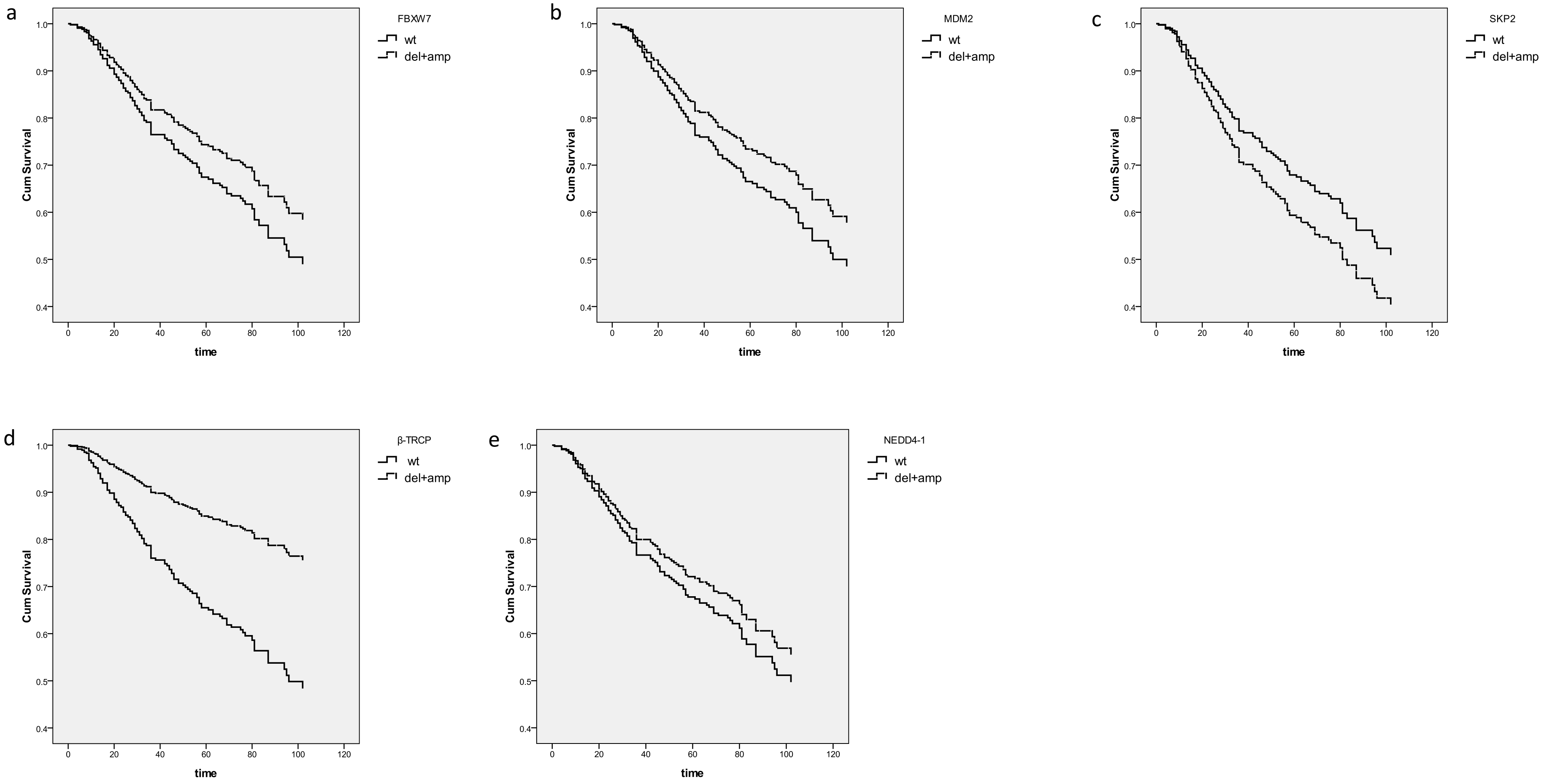

Supplementary Figure S5 Kaplan–Meier curves of overall survival (OS) according to the five genes CNVs in patients with colon cancer. a *FBXW7* CNVs in colon cancer; b *MDM2* CNVs in colon cancer; c *SKP2* CNVs in colon cancer; d *β-TRCP* CNVs in colon cancer; e *NEDD4-1* CNVs in colon cancer.

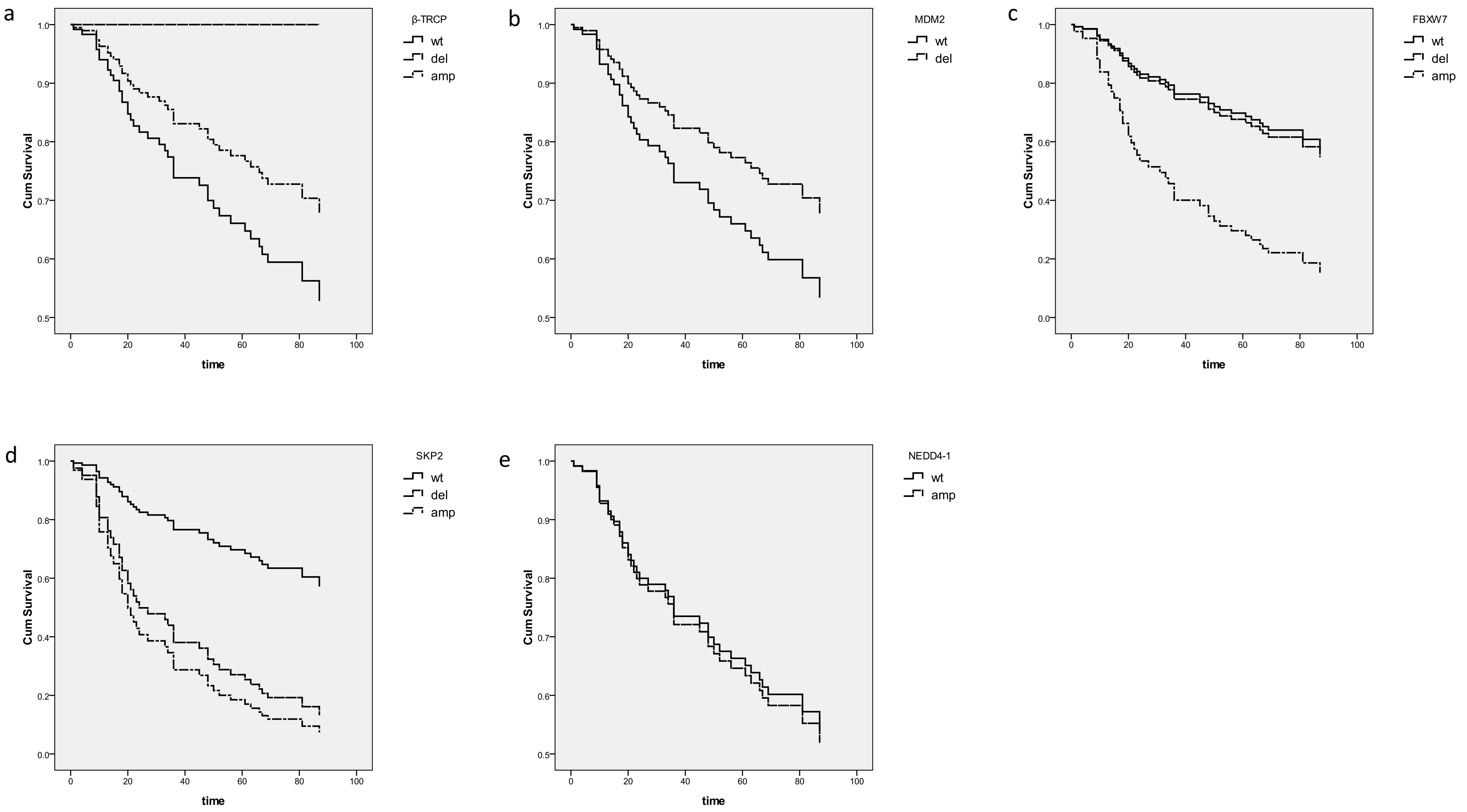

Supplementary Figure S6 Kaplan–Meier curves of overall survival (OS) according to the five genes amplifications in the combined model in patients with colon cancer. a *FBXW7* CNVs in colon cancer; b *MDM2* CNVs in colon cancer; c *SKP2* CNVs in colon cancer; d *β-TRCP* CNVs in colon cancer; e *NEDD4-1*

CNVs in colon cancer.

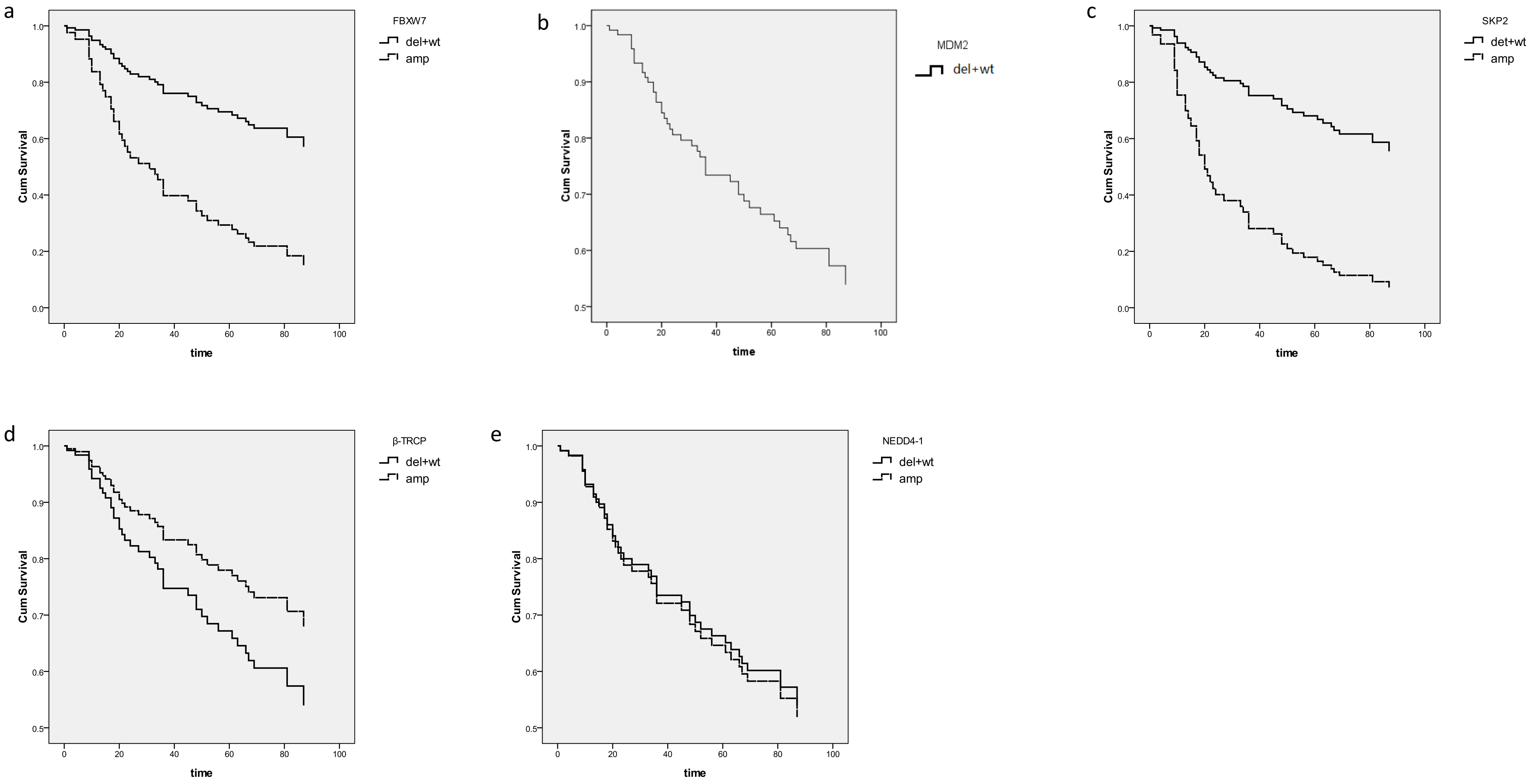

Supplementary Figure S7 Kaplan–Meier curves of overall survival (OS) according to the five genes CNVs in combined model in patients with colon cancer.

a *FBXW7* CNVs in colon cancer; b *MDM2* CNVs in colon cancer; c *SKP2* CNVs in colon cancer; d *β-TRCP* CNVs in colon cancer; e *NEDD4-1* CNVs in colon cancer.

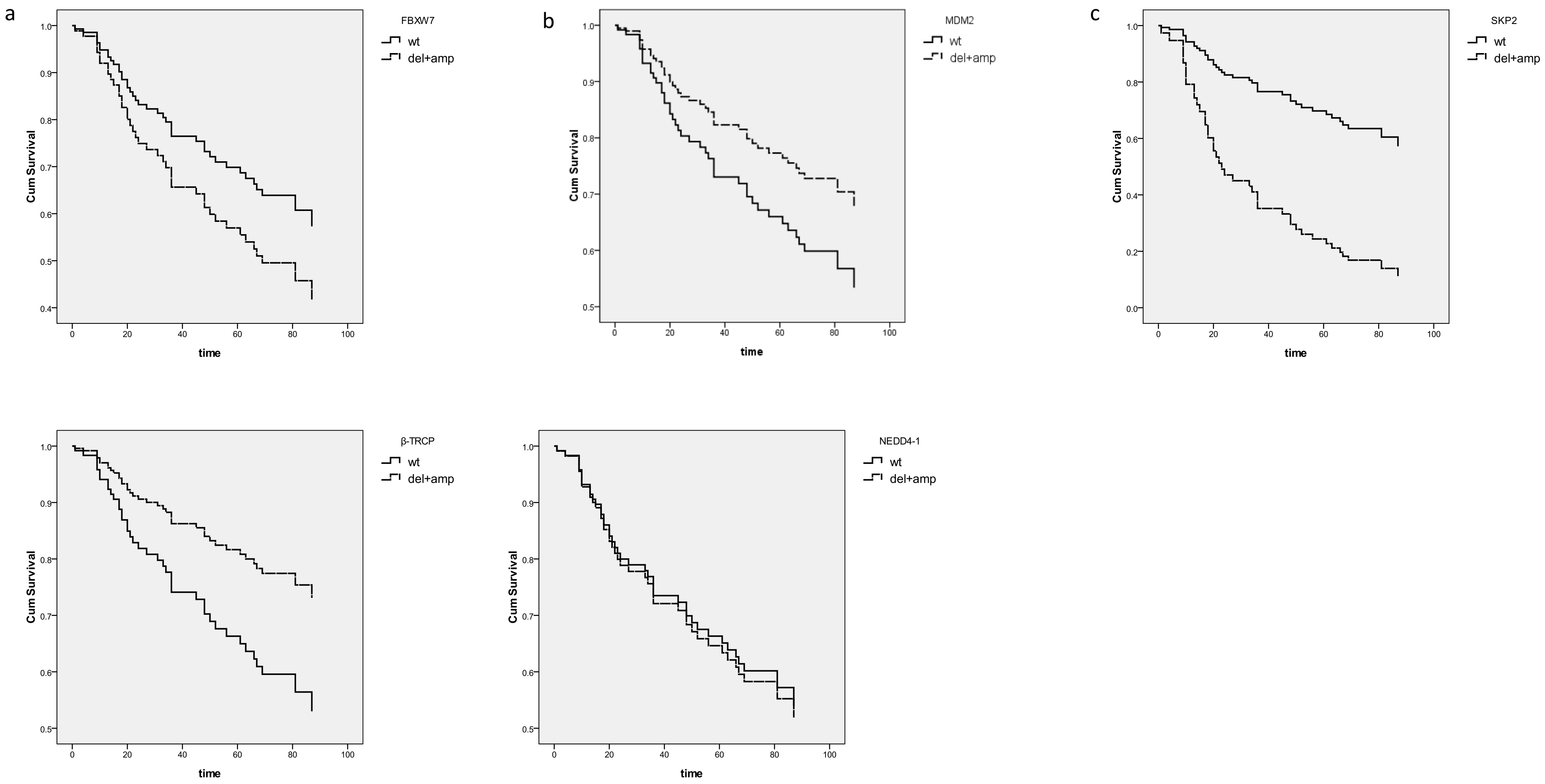

Supplementary Figure S8 Kaplan–Meier curves of overall survival (OS) according to the five genes CNVs in patients with colon cancer. a *FBXW7* CNVs in rectal cancer; b *MDM2* CNVs in rectal cancer; c *SKP2* CNVs in rectal cancer; d *β-TRCP* CNVs in rectal cancer; e *NEDD4-1* CNVs in rectal cancer.

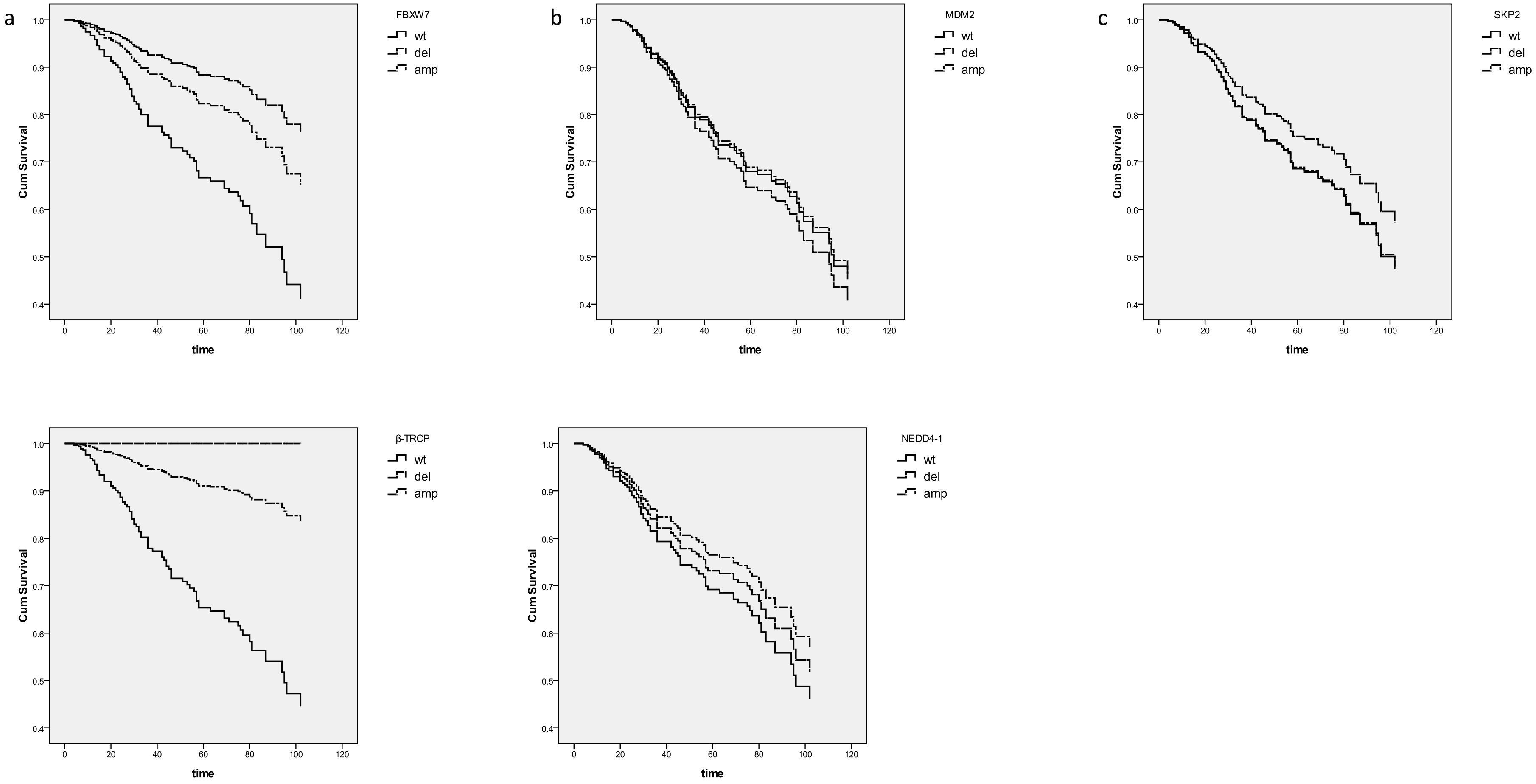

Supplementary Figure S9 Kaplan–Meier curves of overall survival (OS) according to the five genes amplifications in the combined model in patients with rectal cancer. a *FBXW7* CNVs in rectal cancer; b *MDM2* CNVs in rectal cancer; c *SKP2* CNVs in rectal cancer; d *β-TRCP* CNVs in rectal cancer; e *NEDD4-1* CNVs in rectal cancer.

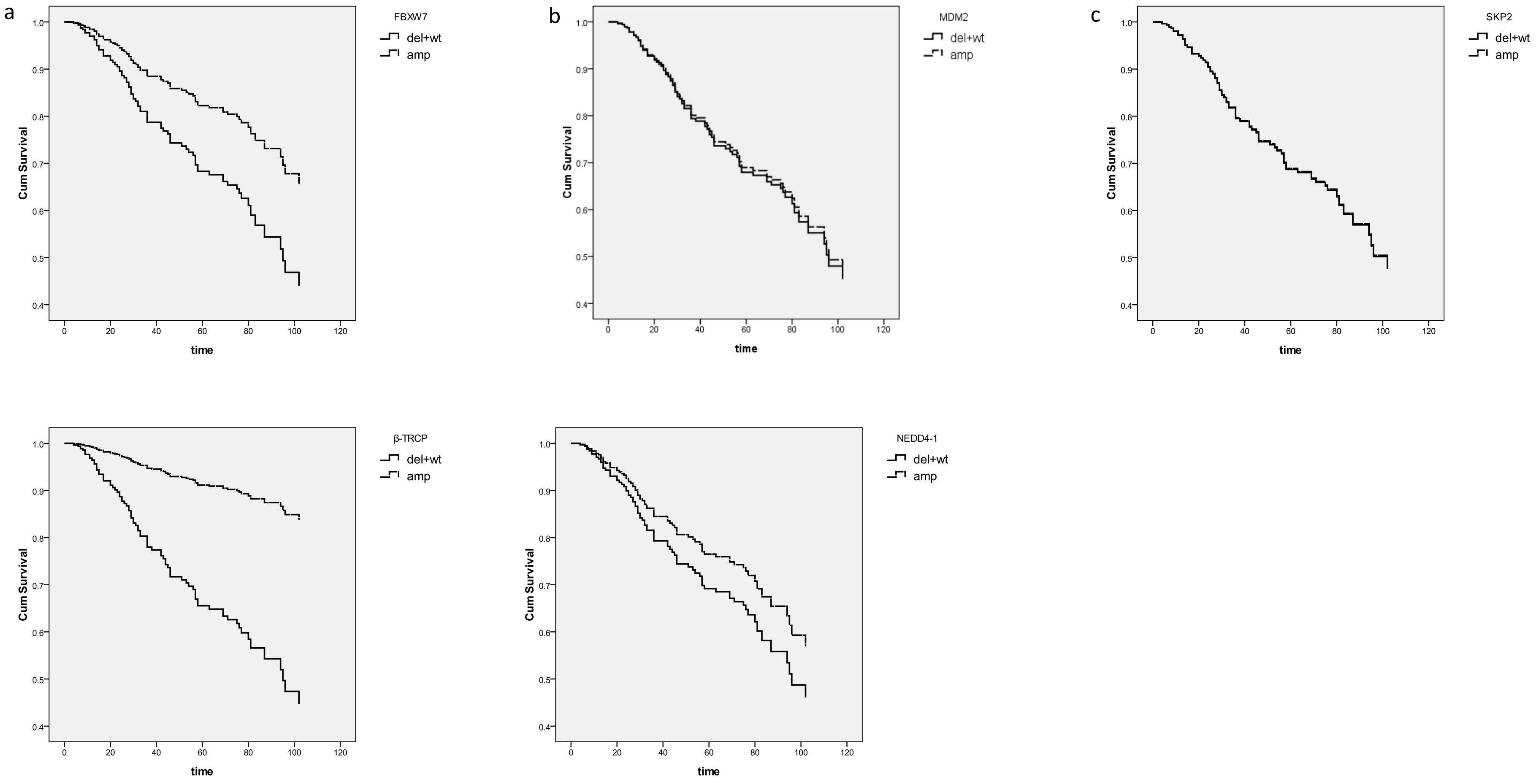

Supplementary Figure S10 Kaplan–Meier curves of overall survival (OS) according to the five genes CNVs in combined model in patients with rectal cancer. a *FBXW7* CNVs in rectal cancer; b *MDM2* CNVs in rectal cancer; c *SKP2* CNVs in rectal cancer; d *β-TRCP* CNVs in rectal cancer; e *NEDD4-1* CNVs in rectal cancer.

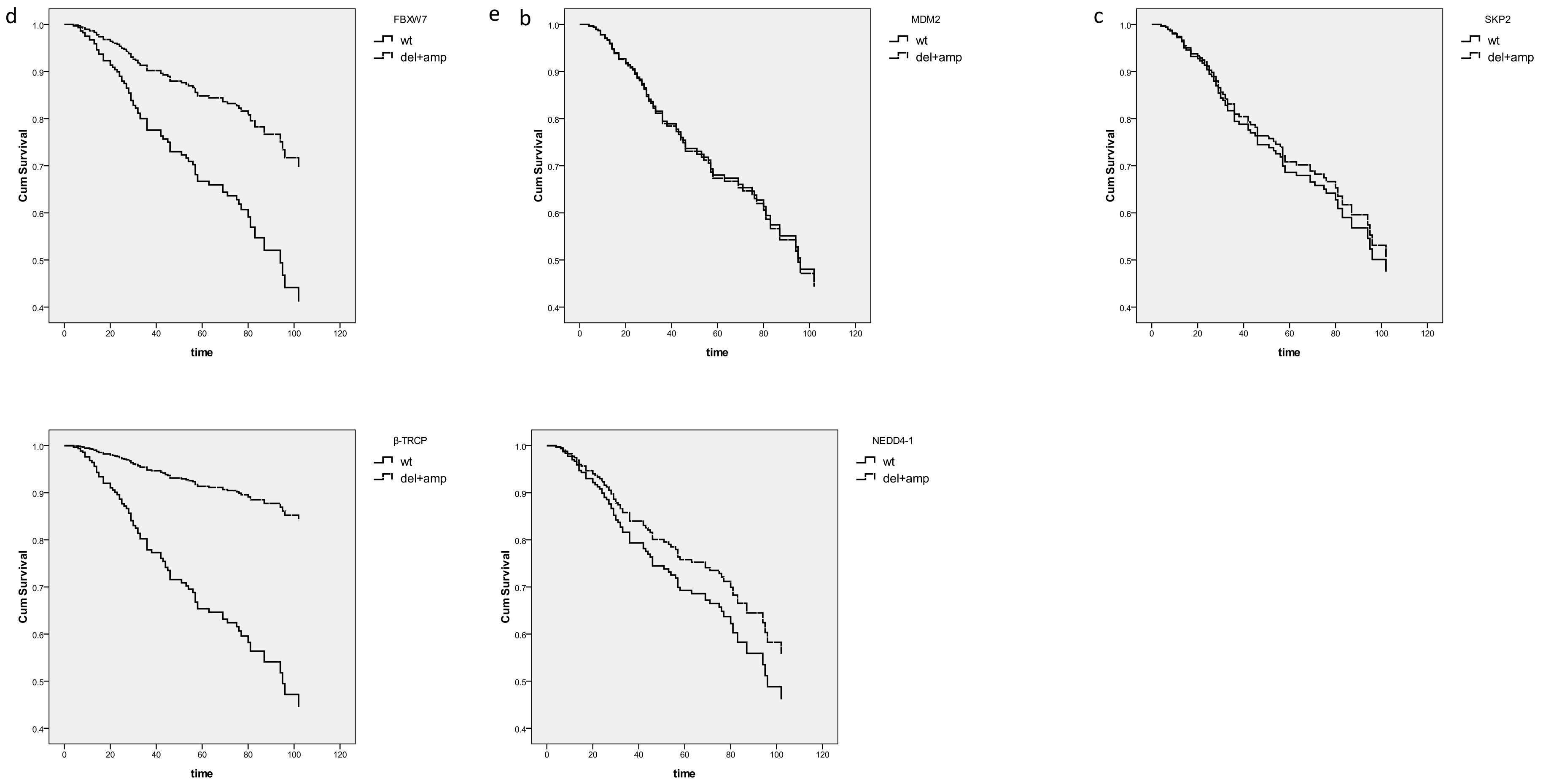

Supplement: Supplementary Information [file srep29869-s1.pdf]
